# Inequitable and heterogeneous impacts on electricity consumption from COVID-19 mitigation measures

**DOI:** 10.1016/j.isci.2021.103231

**Published:** 2021-10-07

**Authors:** Jiehong Lou, Yueming (Lucy) Qiu, Arthur Lin Ku, Destenie Nock, Bo Xing

**Affiliations:** 1 School of Public Policy, University of Maryland College Park, MD 20742, USA; 2 Department of Engineering and Public Policy, Carnegie Mellon University, Pittsburgh, PA 15213, USA; 3 Department of Forecasting, Resource Planning and Development, Salt River Project, Phoenix, AZ 85072, USA

**Keywords:** energy resources, energy policy, energy management, energy Systems, research methodology social sciences

## Abstract

The COVID-19 pandemic has exacerbated energy insecurity and economic hardship among vulnerable populations. This paper provides robust empirical evidence of the degree to which COVID-19 mitigation measures, especially the mandates of school closure and limiting business operations, have impacted electricity consumption behavior in low-income and ethnic minority groups in the United States. We use a regression discontinuity design applied to individual-consumer-level high-frequency smart meter data in Arizona and Illinois to highlight the disparities in mitigation measure impacts. We find that the mandates of school closures and limiting business operations increase residential electricity consumption by 4–5%, but reduce commercial electricity consumption by 5–8%. Considerable heterogeneity is observed across income and race: low-income and ethnic-minority populations experience a larger electricity consumption increase, reflecting the disproportionate impact of COVID-19 on electricity insecurity in the residential sector. Policies that address energy insecurity, especially during the pandemic, become essentially important.

## Highlights

• The COVID-19 measures have exacerbated energy insecurity in the United States
• Electricity consumption increased by 4–5% in the residential sector
• Electricity consumption decreased by 5–8% in the commercial sector
• Low-income and ethnic minority populations experienced a larger impact

## Graphical abstract

**Figure:**
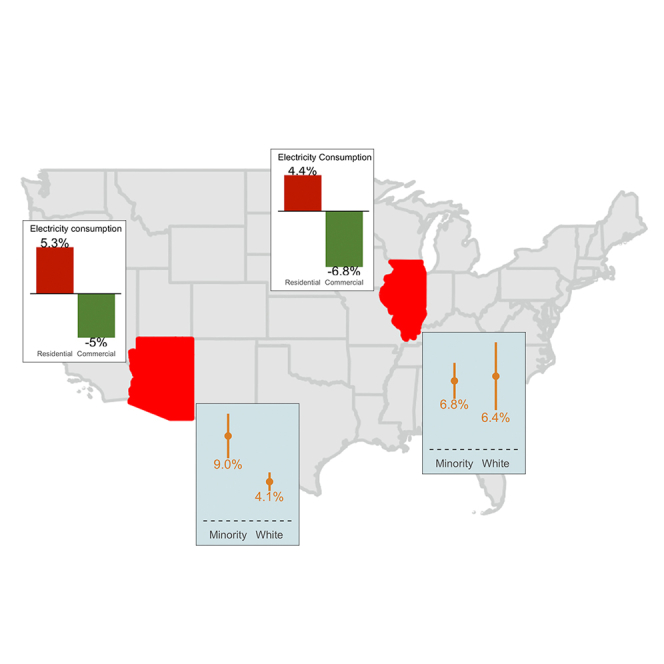

## Introduction

Energy insecurity is defined as the inability to adequately meet the basic energy needs of a household, such as heating, cooling, and cooking. Energy insecurity has been exacerbated in vulnerable populations during the COVID-19 pandemic (Drehobl et al., 2020; Hernández, 2016; Memmott et al., 2021a). In 2015, the US Energy Information Administration (EIA) estimated that, out of 118.2 million US households, 25 million households had to make serious trade-offs between meeting their basic needs, including food and medicine, and paying their energy bills (US Energy Information Administration, 2018). Low-income and ethnic minority households are at greater risk of sinking into energy poverty (Bednar and Reames, 2020; Graff and Carley, 2020) because of spending higher proportions of their income on their energy bills (Hernández, 2016; US Energy Information Administration, 2018). Out of 10 households who had to make concessions on their basic needs in order to cover the cost of their energy bill, it is estimated that four of them would be Hispanic or Black households (Drehobl and Ross, 2016). Although economic expenditures have dominated the energy poverty assessment landscape, energy poverty is multidimensional. It can be driven by the physical conditions of houses, household energy consumption, and energy-related behavior and strategies (Drehobl and Ross, 2016; Hernández, 2016). This poses another layer of social and economic consequence beyond general economic poverty, because the inability to secure stable energy services is correlated with continuous poverty (Bohr and McCreery, 2020), vulnerability to health, and wellbeing (Hernández and Siegel, 2019). Continuous poverty means that despite the consequence of financial poverty, energy insecurity could contribute to future financial poverty as well. Energy insecurity can lead to a series of nutrition, health and education consequences, and thus reduce a household's future income or increase a household's future expenses.

The novel coronavirus, COVID-19, and mandates to mitigate its spread pose a new threat to energy insecurity (Graff and Carley, 2020) for households. By November 21, 2020, over 20 million jobless Americans claimed unemployment benefits across all state and federal programs (U.S. Department of Labor, 2020a; U.S. Department of Labor, 2020b). During this period, the unemployment rate hit a record high of 14.8%, which is substantially greater than the 10.6% unemployment rate of the Great Recession (Falk et al., 2021; Kochhar, 2020). A growing number of states, counties, and cities in the United States began to issue policies to mitigate the spread of the virus and protect public health, such as school closings, reducing business operation hours, the closure of non-essential businesses, and stay-at-home orders (i.e., shelter-in-place orders). Large uncertainty regarding how these exogenous changes would impact electricity demand patterns and the energy burden felt by low-income communities loomed over regions. With energy costs being the highest monthly expense after food for most low-income households (Velody et al., 2003), the financial strain, the new pattern of working from home, and shift to an online education system, combined with the psychological uncertainty of job security during an ongoing pandemic, put energy-poor households at a high risk of economic insecurity and health issues. Under the current COVID-19 pandemic, there is a pressing need to ensure the well-being of the vulnerable groups in regard to reducing energy insecurity, while minimizing the risk of virus spread and helping regions recover from the economic hardships induced by the pandemic. The ability of a region to recover economically from the pandemic will be affected by the residents' ability and willingness to pay for goods and services in a region, and the number of jobs in that region. These factors are directly impacted by the energy sector. Literature focused on regional recovery mentioned that COVID-19 forces most households to use their savings (Martin et al., 2020), which further increases the energy insecurity for vulnerable families. If residents spend higher amounts of money meeting their energy needs then they are less likely to support businesses outside of their critical needs. However, factors, such as employment, secondary and tertiary industries, and revenue generated from small businesses are all influential factors contributing to a region's recovery (Gong et al., 2020). This necessitates the need for researchers to understand the new energy consumption patterns resulting from the pandemic and associated policies in both the residential and commercial sectors.

Current studies have shown that the COVID-19 has a greater impact on energy insecurity for low-income, especially Black and Hispanic, households (Memmott et al., 2021b). However, most of the current studies of COVID-19's impact on electricity consumption rely on aggregated market data or surveys (Abu-Rayash and Dincer, 2020; Agdas and Barooah, 2020; Chen et al., 2020; Edomah and Ndulue, 2020; Fezzi and Fanghella, 2020; Memmott et al., 2021b). The only study, where high-frequency electricity data is analyzed, is Snow et al. (Snow et al., 2020) for Australian households; however, this study fails to analyze heterogeneous impacts across different households (i.e., minority, low-income) due to a lack of demographic data (Snow et al., 2020). This misses an opportunity to unveil how energy insecurity has changed for the poorest members of society. The novelty of our work is providing empirical evidence for the change in energy insecurity based on actual household electricity consumption data. This is critical for policymakers to evaluate how pandemic mitigation policies and demand-side-management practices such as time-of-use (TOU) pricing may impact energy expenditures across different socio-demographic groups, and help them adopt the most adequate measures for reducing energy insecurity. Our study investigates the consumer-level change in hourly electricity demand patterns because of the coronavirus crisis of residential consumers across different income levels and ethnicities using statistical and econometric techniques.

In addition to residential hardships, COVID -19 mandates have imposed unprecedented challenges to small businesses. These enterprises are more vulnerable than their large corporation counterparts due to the financial fragility caused by demand reductions (Bartik et al., 2020; Fairlie, 2020a, 2020b). Short-term disruptions from the COVID-19 mandates can deepen the financial fragility of these small businesses in the long term. If the mandate returns due to additional variants being seen across the globe, low-income and ethnic minority groups may continue to experience hardships during COVID-19 owing to the interruption of small business operations and rising unemployment, which will further impact energy insecurity seen in the residential sector. Therefore, quickly identifying the most impacted small businesses is essential for policymakers to allocate their subsidies equitably.

Evidence shows that when the government distributed COVID relief financial subsidies to small businesses, the most impacted and most vulnerable small businesses were not able to obtain the subsidies because of a lack of the capacity and experience when compared to their larger counterparts (Bartik et al., 2020; Liguori and Pittz, 2020; Hamilton, 2020). Electricity is essential to business operations; policymakers could use changes in electricity consumption patterns to quickly identify the small businesses with the greatest short term impact from the initial COVID-19 lockdowns. For example, small businesses that need the most help might be the ones that had a greater percent decrease in electricity consumption than their counterparts in the same industry. As with any policy, the government needs to establish a set of safeguards to avoid the moral hazard risk, such as validating the actual business status, penalizing bad behavior, or regulatory monitoring. Moral hazard, which originated from the insurance literature, is commonly defined as “lack of incentive to guard against risk where one is protected from its consequences, e.g., by insurance” (Marshall, 2008; Rowell and Connelly, 2012). Moral hazard is fundamentally based on asymmetric information. Thus, we acknowledge that regulatory monitoring and enforcement, even with rigorous measurement, can still present challenges given asymmetric information between firms and the regulator. Although previous studies have analyzed different patterns of electricity usage across various business types (Harder, 2020), these studies primarily relied on descriptive raw data. To our knowledge, our paper presents robust evidence based on the empirical evidence from econometric models to analyze the impact of COVID-19 on electricity consumption patterns across different types of businesses.

In this study, we examine an important component of energy insecurity through electricity consumption behaviors. We focus on electricity consumption, as opposed to natural gas, because of COVID-19 mitigation mandates beginning in the spring and the majority of electricity consumption impacts happening with air conditioning usage for the time period in our analysis. High-frequency electricity data is ideal to study the short-term impact of the COVID-19 mandates and their influence on certain aspects of society owing to the strong linkage between electricity consumption and economic activities (Fezzi and Fanghella, 2020; Stern, 2015). We leave the analysis of COVID's impact on energy usage from other energy types (i.e., gas and oil sector) to future work. We assess the treatment effects of the COVID-19 related mandates on consumers' electricity consumption behaviors in two states, Arizona and Illinois, using two rich high-frequency smart meter datasets on electricity consumption. Arizona (warmer climate) and Illinois (colder climate) represent two very different climate zones, helping us generalize the implications of our results for broader geographical areas. Two sets of COVID-19 mandates are prominent during our study: the school closures and mandates to reduce or close business operation hours. Thus, mitigation efforts in this paper refer to both mandates. Our guiding hypothesis is that the COVID-19 measures increase electricity usage in the residential sector, while simultaneously decreasing electricity usage in the commercial sector. Owing to factors such as living in energy inefficient homes and larger household sizes, the increase in electricity usage will be disproportionately larger for low-income and ethnic minority groups. Thus, we hypothesize that electricity usage increases in the residential sector due to the COVID-19 measures will increase energy insecurity among low-income and ethnic-minority groups because they will be required to spend a larger percentage of their income satisfying their energy needs.

Our individual-consumer-level dataset includes 7,004 residential and 23,117 commercial accounts in Arizona, and 40,771 residential and 40,757 commercial accounts in Illinois from January 1, 2019 to April 30, 2020. We estimate the hourly change in electricity consumption for both residential and commercial users because of these mandates. The analysis controls for possible confounding factors by using a regression discontinuity in time (RDiT) design where residential and commercial electricity consumption is compared before and after the start of the mitigation mandates within a short time window. We further analyze the heterogeneous short-term impacts among different income and ethnic groups of residential consumers, and different industry sectors and sizes of business consumers. We find that low-income and ethnic-minority populations experience the largest residential electricity consumption increase compared to high-income and white populations. We also observe another set of heterogeneous impacts in the commercial sector where larger negative impacts are observed for small businesses in certain sectors. Our results are robust to a set of robustness checks, including a modified difference-in-difference (DID) analysis, a local two-step event study, and a pseudo RD check.

Our results suggest timely measures to address the energy insecurity and economic hardships of the most vulnerable groups during the current and potential future similar crises. The magnitude of energy insecurity has been underestimated due to a lack of formal recognition from the federal government in the United States (Bednar and Reames, 2020). Thus, using the COVID-19 as an entry point, we are able to empirically identify and quantify energy insecurity in a visible manner and call for the policies of energy poverty assistance. We are able to identify those whose job security, educational development, and health are closely linked to their ability to satisfy their energy demand. With our results, governments can fundamentally redesign the way they distribute subsidies, assistance payments, and energy-saving programs with careful consideration of moral hazard risks as the world adapts to international pandemics and increased remote work policies.

## Results

### Effect of COVID-19 on the residential sector

The RDiT approach (see STAR Methods) uses a narrow window around the enforcement date of each policy to identify the COVID-19 mandates' effects on the change in electricity consumption, and uses flexible polynomials of time to control for other unobserved time-varying confounding variables that can influence electricity consumption. The RDiT method delivers the local average treatment effect at the moment when the policy is implemented. We focus on the local treatment effect seen in the first hour or day of the mandate because the presence of the immediate treatment effect (e.g., increase in energy disparity) implies the likelihood of longer-term negative policy impact. In addition, our modified difference-in-differences (DID) analysis investigates the treatment effects during a longer time period following the policy implementation (see Tables S7–S9). Thus, our work allows policymakers to understand both the immediate and long-term impacts of environmental shocks (i.e., COVID pandemic) on different members of the population.

**Figure 1:**
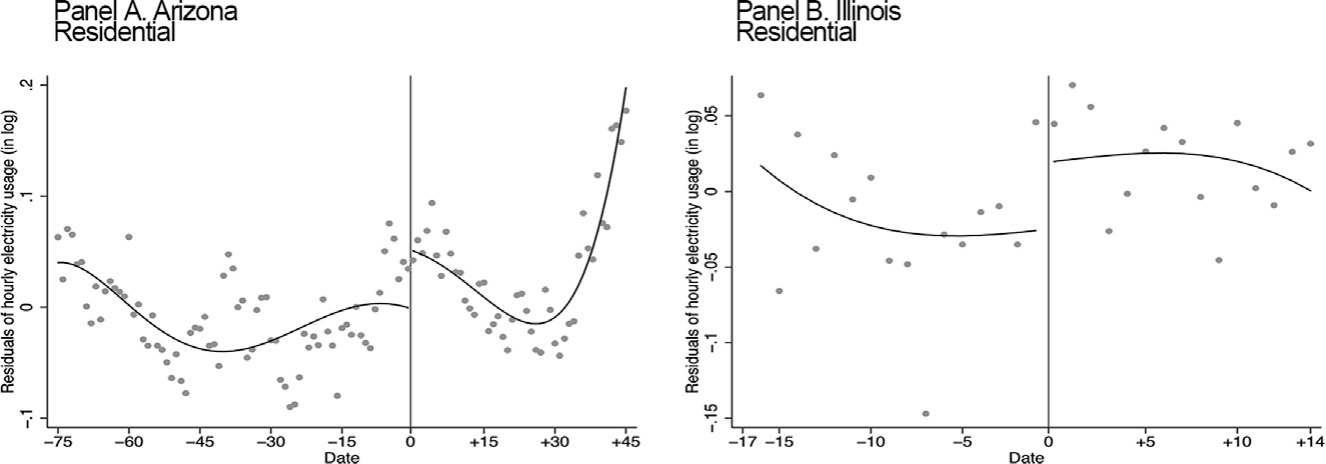
Residential daily averaged hourly electricity consumption percentage change in Arizona and Illinois The black circles are daily averaged hourly residuals of log electricity consumption, averaged across all hours of the day (after controlling for covariates, such as weather, the hour of the day, day of the week, the month of the year) for both Arizona and Illinois. The black fitted lines are values obtained from regressing the residuals on the mitigation mandate dummies. Because of the short gaps between the days of these two mandates, we adopt a fifth-order polynomial on the date for the school-close mandate in Arizona, and a third-order polynomial on the date for the School-close mandate in Illinois. In both states, school-close orders were enforced in the morning so we use the same day as the threshold. The polynomial orders are chosen by the Bayesian Information Criterion (BIC), and we also reported results for other polynomial orders in Table S10. The different numbers of days in the x axis for AZ and IL are due to the different datasets of the two states. In AZ our dataset covers 4 months, while in IL the data spans one month.

In each state, we set the threshold based on school closures and business operation restrictions in 2020. In Arizona, the mandates instituting school closures and reduced business operation hours for our metropolitan region both happened on March 16^th^. In Illinois, the school closures began on March 17^th^. The estimated effect of the school and business closure mandates on electricity consumption in the residential sector is shown in Figure 1. Panel A illustrates the residuals of log electricity consumption (after controlling covariates and polynomials of time) in Arizona within a four-month window. Panel B plots the residuals in Illinois within a one-month window. Illinois only has one month because we only have the panel data with identifiable individuals for one month in March (see STAR Methods). Both Panel A and Panel B depict that residential users increase electricity consumption due to the school closure in both states. When school closes, kids and parents need to stay home for a longer time and thus increase energy consumption.

We report the main RDiT estimates for the effects of the mitigation measures on residential electricity consumption in Table 1. The point estimates in columns (1) and (2) indicate that the school closure mandate increases the hourly electricity consumption by 5.3% in Arizona and 4.4% in Illinois. Both results are statistically significant at the 1% level. The polynomial orders presented in the table are chosen by the Bayesian Information Criterion (BIC) (for the results using other polynomial orders see Table S10). Across all polynomial orders, the estimate of the effect of the COVID-19 measures on electricity consumption stays relatively stable, making our results reliable and robust. Additionally, we report estimated treatment effects from the local linear specification in columns (3) and (4) with different local bandwidths of the COVID-19 measure discontinuity. The local estimation serves as a robustness check for our main results from the global specification. In classic RDiT literature, it is a standard finding that results from the global polynomial being more precise than the local linear model (Hausman and Rapson, 2018).

**Table 1:** Regression discontinuity estimates of changes in residential electricity usage (log) due to COVID-19 mandates: global polynomial results

	(1)	(2)	(3)	(4)
	Arizona (Global)	Illinois (Global)	Arizona (Local)	Illinois (Local)
	School closure	School closure	School closure	School closure
**COVID-19 Mandates**				

	0.053 ∗∗∗	0.044 ∗∗∗	0.013 ∗∗∗	0.031 ∗∗∗
	(0.004)	(0.002)	(0.003)	(0.002)
Weather-related variables	Yes	Yes	Yes	Yes
Month FE	Yes	No	Yes	No
Day-of-week FE	Yes	Yes	Yes	Yes
Holiday FE	Yes	No	Yes	No
Hourly FE	Yes	Yes	Yes	Yes
Account FE	Yes	Yes	Yes	Yes
Observations	19,998,526	30,283,001	4,962,531	8,793,256
Number of households	7,004	40,771	7,004	40,771

Notes: 1. Weather-related control variables include temperature (in a restricted cubic spline format), precipitation (linear and quadratic format), air pressure, relative humidity, and wind speed.

Standard errors, clustered by account id, are in parentheses.

2. Columns (3) and (4) are local linear approaches to validate the results from the polynomial approach. In this setting, a narrower bandwidth of 15 days before and after the policy effective dates (for Arizona) and 4 days (for Illinois) are adopted. We adopted different bandwidths for Arizona and Illinois because of the different periods between the school closure day and stay-at-home order date (AZ – 15 days, IL – 4 days). We use different bandwidths for the local linear regression because we do not want to include the impact of the stay-at-home order in the evaluation of the impact of the school closure date. Additionally, we conducted robustness checks with different bandwidths of the local RD models in Table S11.

∗∗∗ Significant at the 1% level. ∗∗ Significant at the 5% level. ∗ Significant at the 10% level.

Several factors can help explain the overall trend of increased electricity consumption in both states. First, the increased number of residents staying at home due to the school-closure mandates caused higher overall electricity consumption. A recent survey reports that nearly 93% of households with school-age children were involved in remote learning from home (US Census Bureau, 2020). Second, cooking and lighting might contribute to increased electricity consumption. Based on a survey conducted in Australian households during the COVID-19 lock-down, researchers found that energy usage for cooking increased significantly among almost all households (Snow et al., 2020). Especially in Arizona, the school closure mandate along with the restricted business operation mandate on the same date “forced” households to cook more frequently compared to the pre-lockdown phase because all restaurants were either closed or only offered take-out (Hunter, 2020).

In Arizona and Illinois, the state-wide stay-at-home orders occurred after the school closure mandates meaning the electricity consumption behavior would have already seen a shift due to more children, and adults with reduced working hours, and being at home. Although most of the literature focused on the impact of stay-at-home order, our research reveals that the electricity consumption pattern of COVID-19 previously shifted because of the earlier mandates (i.e., school closures and the reduced business operations). We provide the stay-at-home order results in Supplementary Section S1, but note that they are most likely confounded by the behavior changes already induced by the school and business closure mandates.

### Heterogeneous effects by income and ethnic groups

We now examine energy insecurity in the following three dimensions: racial, income, and energy burden. Our results are important for understanding the energy security implications of COVID mitigation measures and might contribute to the broader energy equality discussion.

**Figure 2:**
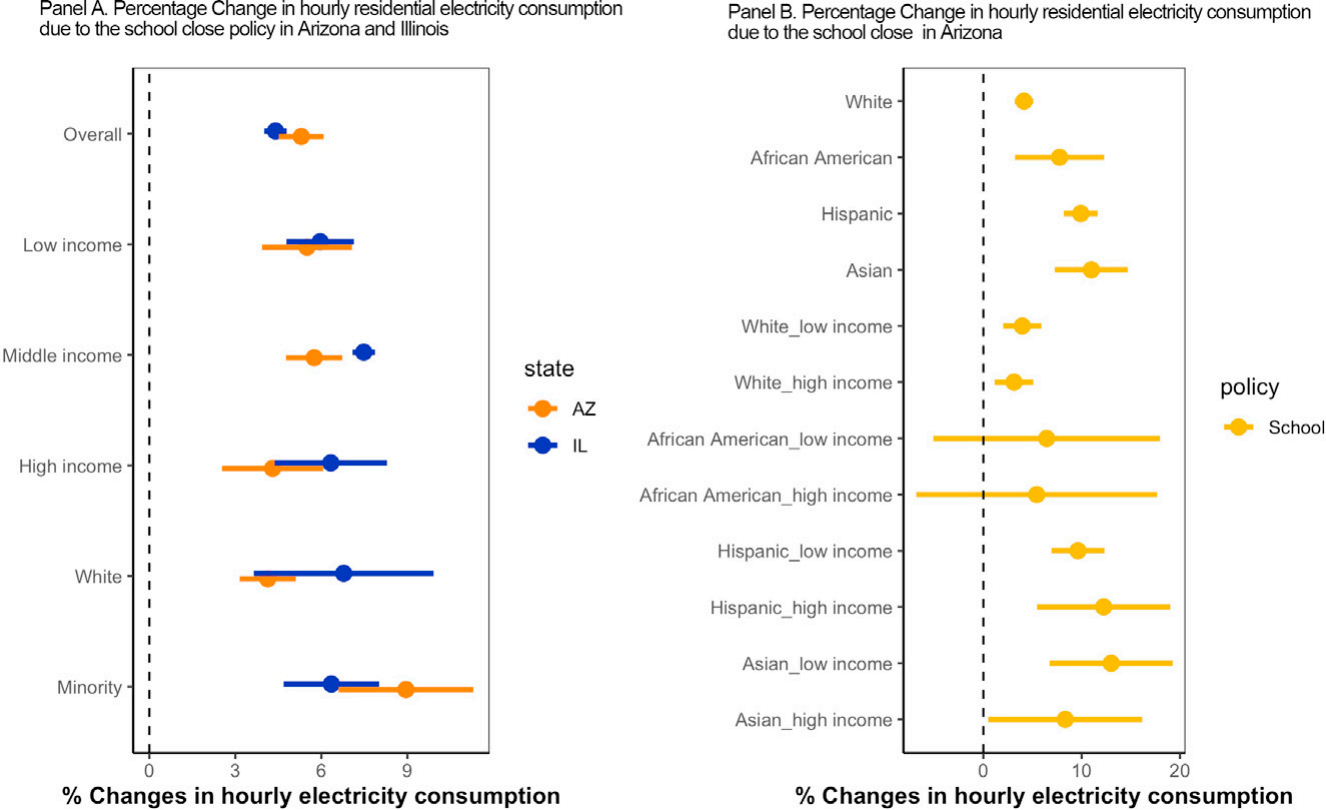
Percentage change in daily averaged hourly residential electricity consumption because of the implementation of the school closure/restricted business operation by different demographic groups The colored dots represent the percentage of changes in hourly electricity consumption, which are obtained from running the RDiT specification separately. The colored horizontal bars represent the 95% confidence intervals of the estimations.

First, we show that there is an increase in energy insecurity among ethnic-minority households. Panel A in Figure 2 presents the estimates of the impact from the COVID mitigation measures by running separate RDiT models on different demographic groups of residential consumers. The estimates show that ethnic-minority consumers (i.e., non-white consumers, which primarily covers Africa American, Hispanic, and Asian consumers) have a greater percentage increase in electricity consumption compared to the white population in Arizona due to the school closure mandate. Although there is no statistical difference of the increased percentage in Illinois between ethnic-minority consumers and the white population, we still observe increased energy insecurity in both groups of population. The disparate effect is even larger in Arizona because of its large proportion of the Hispanic population who have on average larger household sizes. Panel B in Figure 2 shows that Asian consumers have the largest percentage increase in electricity consumption because of the school closure, followed by the other minority groups (Hispanic, Black, etc.). For example, in Arizona, after the school closure mandate, the electricity consumption in the low-income non-white population increases by 9.69% (Black: 6.44%, Hispanic: 9.63%, and Asian: 13.00%), whereas electricity consumption in the low-income white population increases by 3.96%.

Existing studies have found a similar electricity consumption pattern between the white and ethnic minority group in the pre-COVID-19 era, partially because low-income ethnic-minority households reside in less efficient homes (Graff and Carley, 2020; He et al., 2020) and also have larger household sizes (See household characteristics information in Tables S17–S19, and Figure S2). Figure S2 shows that minority groups have larger household sizes (number of household members) compared to the white population (3.2 per house of low-income Hispanic family vs 2.1 per house of low-income White family, 2.7 per house of high-income Hispanic family versus 2.1 per house of high-income White family). Overall, larger household sizes and energy inefficient homes (shown by house age in Figure S2) explain why there is a greater percentage increase in electricity consumption of minority consumers during COVID mitigation measures. The increases in electricity consumption combined with the lack of energy-efficient housing features (Cong et al., 2021; Drehobl et al., 2020; Drehobl and Ross, 2016; Reames, 2016a) and larger household sizes also suggest that the low-income and ethnic minority groups are more vulnerable to energy poverty and insecurity during the COVID-19.

Second, we show that there is an increase in energy insecurity among low-income households. In Figure 2 Panel A, we observe that the low-income population has a higher electricity consumption percentage change compared to the overall population in both states. The high increase in energy means more energy insecurity for the low-income population. In addition, we observe that the increase in electricity consumption in the lower-income groups because of school closure is 1.2 percentage points higher than that of the higher-income groups in Arizona. However, the lower-income groups show no difference in electricity consumption compared to the higher-income groups in Illinois. The results could reflect several possibilities. One possibility of the increased energy consumption in the lower-income groups in Arizona is that they live in energy inefficient homes (Graff and Carley, 2020; He et al., 2020). We find that the average house age of the low-income population is 53.34 years, whereas the average house age of the high-income population is 51.89 years. As older houses tend to consume more energy, the physical condition of the older house could explain the increase in electricity consumption (Kwon and Jang, 2017). Although we are aware that lower-income groups have fewer opportunities to work from home because these groups occupy a large portion of COVID-19 essential businesses (Lou et al., 2020; Wright et al., 2020), we still observe the increased energy insecurity due to the increased electricity consumption caused by the COVID-19 for the low-income population.

Third, we show the energy burden by calculating the percent of income spent on energy (electricity) bills. In Table S21, consumers with different social-economic characteristics show different energy burdens. In Illinois, the energy burden increases from 2.31% to 2.44% for low-income consumers; for high-income consumers, the energy burden increases slightly (0.79%–0.84%). In Arizona, the average energy burden of low-income consumers is two times higher than that of high-income consumers. Within the same income level, Hispanic consumers have a higher energy burden (1.87%) compared to the white population (1.53%). Before COVID-19, the energy burden for low-income Hispanic consumers was 1.71%, compared to 1.47% for low-income white consumers. After the Covid-19 mitigation measures, within the low-income group, the energy burden for Hispanic consumers increased by 0.16%–1.87%, whereas the low-income white consumers only increased from 0.06% to 1.53%. In general, our results show that the low-income and ethnic-minority population has to pay an additional $10 per month becaue of the COVID-19 mandates. Other national-scale studies also confirm that certain socio-economic groups, such as African and Latino Americans, spend significantly more on energy than their white counterparts (Graff and Carley, 2020). What makes our results novel is that this trend expands to extreme events such as the COVID-19 pandemic, and highlighting that this energy insecurity worsens during such disasters. This finding illustrates why certain policies are urgently needed to alleviate the disproportionately negative impacts on these groups. We note that our estimation of the COVID-19 mandate's impacts on energy burden is underestimated because March and April are mild weather months, and the need for space heating or cooling will be greater in the summer and winter. Space heating and space cooling account for 31% of electricity expenditure at the national level (EIA, 2020a). As a result, the full impact of COVID-19 and associated mandates will become exacerbated in summer and winter for low-income ethnic-minority consumers.

Heterogeneous effects are embedded in individual consumers' electricity consumption patterns, as seen in the RDiT regressions conducted for each consumer separately. The individual changes in electricity consumption resulting from the mitigation measures are shown in Figure S10 Panels A and C, which highlight the large variability in mitigation measures' impacts.

Overall, COVID-19 intensified the magnitude of energy insecurity of low-income and ethnic minority groups in both AZ and IL. By combining the factors related to physical house conditions, energy efficiency features, and energy burdens into our analysis, our results present a more comprehensive picture of the COVID-19 mandates on energy insecurity among low-income and ethnic minority populations in the residential sector in Arizona and Illinois.

### The hourly pattern of residential electricity consumption

**Figure 3:**
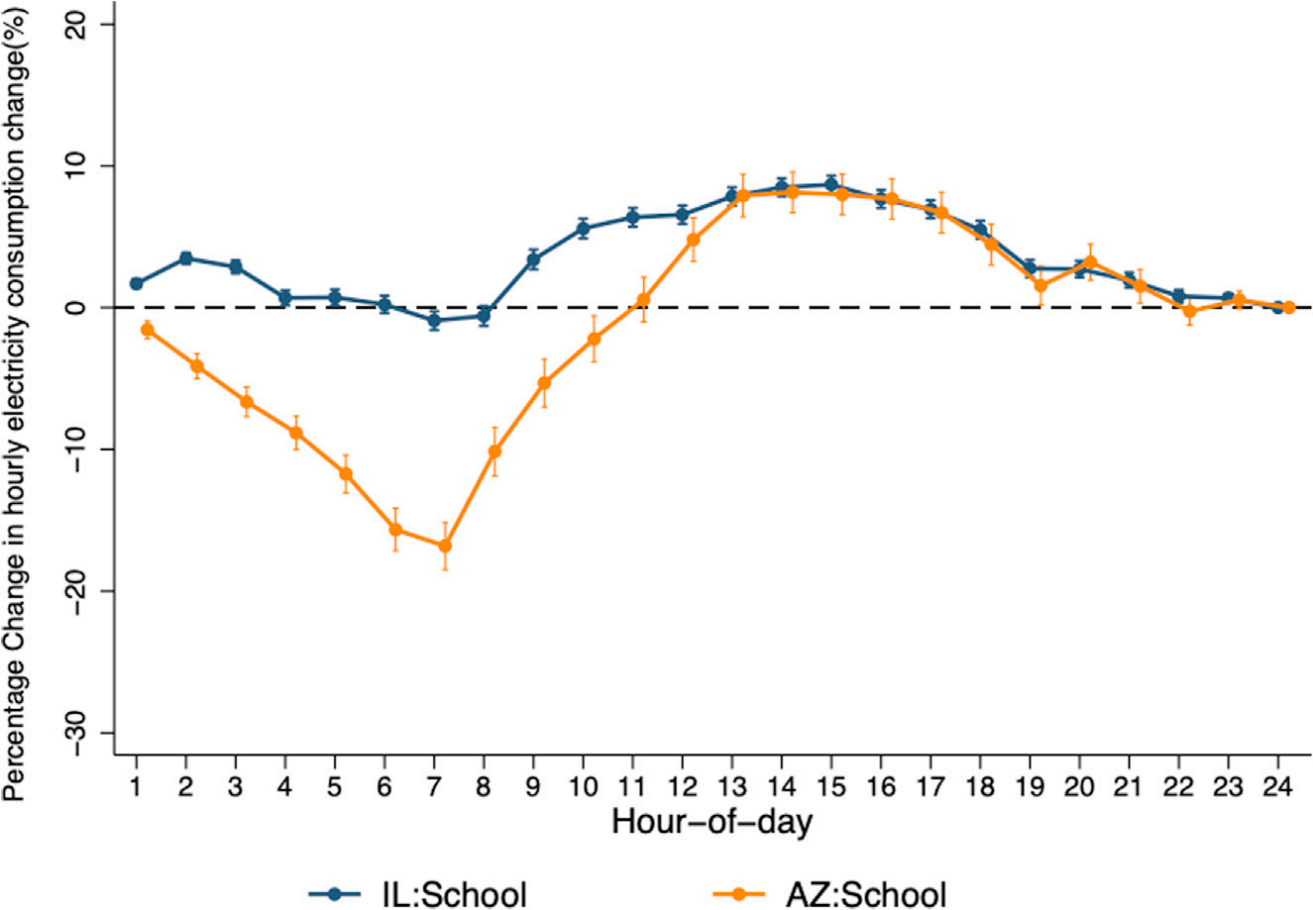
Percentage change in residential hourly electricity consumption (%) because of the implementation of the school closure/restricted business operation The colored dots represent the percentage changes in hourly electricity consumption, which are obtained from the two stage-event study specifications. The colored vertical bars with corresponding colors represent the 95% confidence intervals of the estimations. The results in this figure are obtained from a two-stage event study (see STAR Methods).

Our work unveils how the pandemic mitigation efforts change energy consumption patterns at the aggregate level across the treatment period. In addition to the overall electricity consumption changes, the hourly pattern can indicate how household activities pose behavior shifts, as well as showing how policy changes (e.g., adjusting time-of-use electricity pricing) will affect consumers. Figure 3 illustrates how hourly electricity consumption patterns change following the mitigation measures. In both states when the school closure mandate was implemented, hourly electricity consumption after 11 am increased by at least 4% on average. The morning peak (usually 6 am–10 am) electricity consumption shifts due to the mitigation mandates in both states. Both observations show a delay in electricity consumption in the morning peak hours during the COVID-19 era. The shape of the “camel curve” with two daily humps was reshaped to an extended singular peak during the middle of the day. The shifted pattern provides opportunities and challenges to the utility companies. Challenges stem from the additional monitoring and adjusting for shifting load patterns to make sure of the grid stability. Opportunities arise for the additional renewable sources being added to the grid.

The most convincing evidence should trace to the behavior change in mobility. Figures S3 and S4 depict the percentage of staying at home by the hour of the day. Both mitigation measures lead to a 5% and 10% increase in the number of people staying at home almost every hour during the daytime (9 am–5 pm) compared to the same month last year based on mobility data in Arizona and Illinois, respectively. This partially explains why electricity usage increases even in the midnight hours. The sharp decline of electricity demand in the morning and evening peak hours reflects an underlying pattern: these mitigation measures change people's daily energy behavior and will impact the electricity system fundamentally if remote working becomes more common in the future. Studies already show that the capacity of remote working is 45%, 56%, and 39% in the developed economies, such as the United States, Germany, and Norway, respectively (Alipour et al., 2020; Holgersen et al., 2020; U.S. Bureau of Labor Statistics, 2020a), although a much lower percentage, 23.7% worked remotely before the COVID-19 pandemic in the United States (U.S. Bureau of Labor Statistics, 2020b). The large increase in electricity from 3 pm to 7 pm implies to the policymakers that a time-of-use rate (TOU) that has peak hours with higher electricity prices during these hours might cause an even larger energy burden for vulnerable residential households. In our study region, more than half of the TOU users in Arizona have higher prices during 3-7pm. For a detailed price plan, please see Table S22. Among the 7,004 consumers in our sample in AZ, only 141 consumers switched plans during our study period. Within the 141 consumers, only 18 switched plans after the pandemic. When we calculated the energy burden for TOU consumers (Table S25) we found that the difference in electricity bills between pre-COVID and post-COVID is larger for TOU consumers, and post-COVID is at least 36% larger for the TOU consumers. Thus utilities may need to consider how TOU pricing will need to be adjusted, because the these mandates limit the vulnerable consumers' options to reduce their consumption by going to work or other cooling and heating centers (e.g., grocery stores and libraries) outside of the home.

We further differentiate the residential consumers in Arizona between the TOU and non-TOU consumers and confirmed the different types of behavior change between these two groups. The TOU plans in AZ cover peak hours between 2 pm and 8 pm in wintertime. Owing to the school closure mandate, the increase in hourly electricity consumption of TOU users is higher compared to that of non-TOU users between 2 pm and 8 pm, by an average of 7% (see Figure S11). This is because prior to the mandates TOU consumers were able to have lower energy consumption during 2-8pm by staying away from home. With the mandates, they have to stay at home and thus increase much more electricity consumption during the peak hours compared to non-TOU consumers. In addition, we also observe a similar trend of low-income ethnic-minority households with TOU plans to have a higher increase in hourly electricity consumption because of the school closure mandate compared to the high-income white population (see Table S24). The selection into TOU is not randomly assigned (i.e., consumers self-select) and thus may pose selection bias in econometric estimation. Despite the fact that TOU and non-TOU consumers exhibit different behaviors, our individual-consumer fixed effects in our model control for the unobserved factors that cause the self-selection into TOU plans.

In addition, our results show increased electricity consumption for homes during the day when there is abundant solar irradiance. The abundant solar irradiance implies that installing solar panels at residential properties will help offset more electricity consumption in a work-from-home era compared to the pre-pandemic era when peak residential electricity happens during the early evening hours when solar panels cannot generate much electricity. The mandates might provide new opportunities for deploying more solar panels in the residential sector, but could worsen the energy burden if high-income households are the main consumers adopting this infrastructure. This highlights the need for investment in subsidies and incentives for landlords to adopt this technology.

### Effect of COVID-19 on the commercial sector

**Figure 4:**
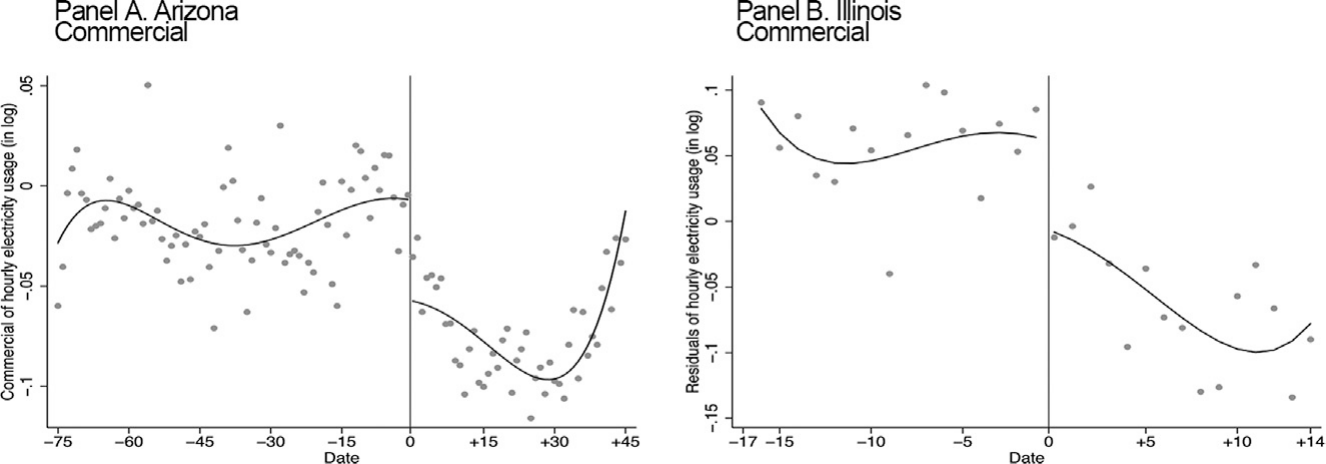
Commercial daily averaged hourly electricity consumption percentage change in Arizona and Illinois Notes: The black circles are daily average hourly residuals of log electricity consumption (after controlling for covariates, such as weather, the hour of the day, day of the week, and month of the year) for both Arizona and Illinois, averaged across all hours of a day. The black lines are the fitted polynomial to model the nature of the treatment through the strategy of regressing the residuals on these mitigation measure dummies. Due to the different durations of the time window of the two states, to avoid overfitting, we adopt a fifth-order polynomial on the date for school closure mandate in Arizona, and a fourth-order polynomial on the date for the school closure mandate in Illinois. We select polynomial orders using the BIC. Results for other polynomial orders are reported in Table S10. The different numbers of days in the x axis for AZ and IL are due to the different datasets of the two states. In AZ, our dataset covers 4 months, whereas in IL, we only have one month of data.

We depict the estimates of the effects of the two COVID-19 mitigation measures on electricity consumption in the commercial sector from the RDiT in Figure 4. Panel A illustrates the residuals of log electricity consumption in Arizona within a four-month window. Similarly, panel B plots the residuals in Illinois within a one-month window. At a glance, both panels A and panel B illustrate that commercial users decrease electricity consumption because of the school closure mandate in both states.

Table 2 illustrates the RDiT estimated treatment effect for commercial electricity consumption. The hourly electricity consumption decreased by 5% in Arizona and 6.8% in Illinois because of the school closure mandate (statistically significant at 1% level). Again, the polynomial orders are chosen by the Bayesian Information Criterion (BIC), and we also reported results for other polynomial orders in Table S10. Across all polynomial orders, the estimates of the effect of the COVID-19 measures on electricity consumption stay relatively stable. We further present the estimated treatment effect from the local specification in columns (3) and (4) with different local bandwidths of the COVID-19 measure discontinuity. The local estimation serves as a robustness check for our main results from the global specification. Overall, we observed a combined statistically significant effect of decreasing electricity consumption due to the implementation of the school closure/reduced business operation measures in both states.

**Table 2:** Regression discontinuity estimates of changes in commercial electricity usage (log) due to COVID-19 mandates: global polynomial results

	(1)	(2)	(3)	(4)
	Arizona (Global)	Illinois (Global)	Arizona (Local)	Illinois (Local)
	School closure	School closure	School closure	School closure
**COVID-19 Mandates**				

	−0.050 ∗∗∗	−0.068 ∗∗∗	−0.027 ∗∗∗	−0.088 ∗∗∗
	(0.002)	(0.002)	(0.002)	(0.002)
Weather-related variables	Yes	Yes	Yes	yes
Month FE	Yes	No	Yes	No
Day-of-week FE	Yes	yes	Yes	yes
Holiday FE	Yes	No	Yes	No
Hourly FE	Yes	yes	Yes	yes
Account FE	Yes	yes	Yes	yes
Observations	61,442,445	30,283,001	9,280,270	81,743,262
Number of businesses	14,271	40,757	14,097	40,757

Notes: 1. weather-related control variables include temperature (in a restricted cubic spline format), precipitation (linear and quadratic format), air pressure, relative humidity, and wind speed.

Standard errors, clustered by account id, are in parentheses.

2. Columns (3) and (4) are local linear approaches to validate the results from the polynomial approach. In this setting, a narrower bandwidth of 15 days before and after the policy effective dates (for Arizona) and 4 days (for Illinois) are adopted. The reason that we adopted different bandwidths for Arizona and Illinois is because of the different periods between the school closure day and stay-at-home order date. In Arizona, it is 15 days, and in IL, it is four days. That's why we use different bandwidths for the local linear regression because we do not want to include the impact of the stay-at-home order in the evaluation of the impact of the school-closure date. Additionally, we conducted robustness checks with different bandwidths of the local RD models in Table S11.

∗∗∗ Significant at the 1% level. ∗∗ Significant at the 5% level. ∗ Significant at the 10% level.

### Heterogeneous effects by industry sectors and sizes

**Figure 5:**
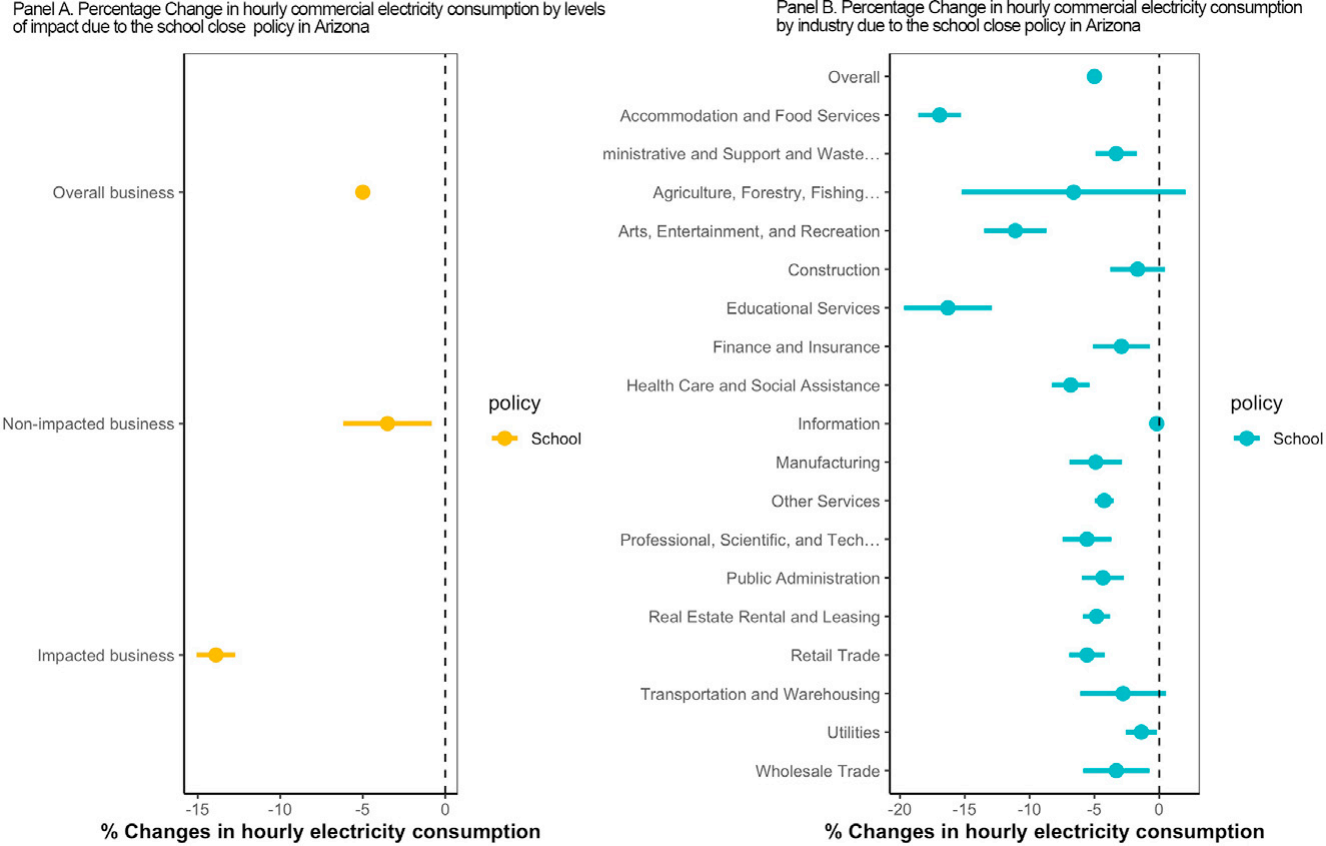
Percentage change in daily averaged hourly commercial electricity consumption because of the implementation of the school-closure/restricted business operation mandate by industry in Arizona The colored dots represent the percentage of changes in hourly electricity consumption, which are obtained from running the RDiT specification separately. The colored horizontal bars with corresponding colors represent the 95% confidence intervals of the estimations. We have dropped the mining sector from the figure due to insufficient observations.

We now examine the heterogeneous effects to identify the most vulnerable businesses from their patterns of electricity consumption change. When firms see a large decline in electricity consumption this could imply a larger loss of business operations and revenues as well as more negative impacts on the income of their employees (Fairlie, 2020a, Hamilton, 2020, Kurmann et al., 2021). We first divide the entire business set into two subgroups (i.e., non-impacted and impacted) based on the severity of the potential impacts on business. The impacted industries in our commercial analysis span retail, education, entertainment, and food services, which is highly correlated to the non-essential business. The remaining industries are categorized as non-impacted business industries. Effects on the non-impacted and impacted businesses are depicted in Figure 5 panel A. Our results show that there is a clear difference across the impacted and non-impacted businesses in terms of electricity consumption. In Arizona, the school and business mandates happened on the same day in the areas that the utility company, SRP, serves. Thus, these impacted industries are unable to perform business because of the school closure order, such as education services, or they have to reduce operating hours and only maintain limited business operations in Arizona, such as retail and food services. Following school closure and reduced business operations mandates, we observe a 15% decrease in electricity consumption in impacted business industries, compared to a 4% decrease in electricity consumption in non-impacted business industries.

Second, as shown in the heterogeneous effects by sectors in Figure 5 panel B, impacts on electricity consumption patterns varied across industries, with accommodation and food services, and education services to be the two sectors with the biggest impact. We observed large declines in accommodation and food services (16.94%), education services (16.30%), arts, entertainment and recreation (11.10%), retail trade (5.57%), and wholesale trade (3.31%) due to their inability of moving to remote operations as well as reduced demand of their products and services. This could also imply that employees and business owners in these industries might experience a larger negative impact on their income and levels of unemployment.

**Figure 6:**
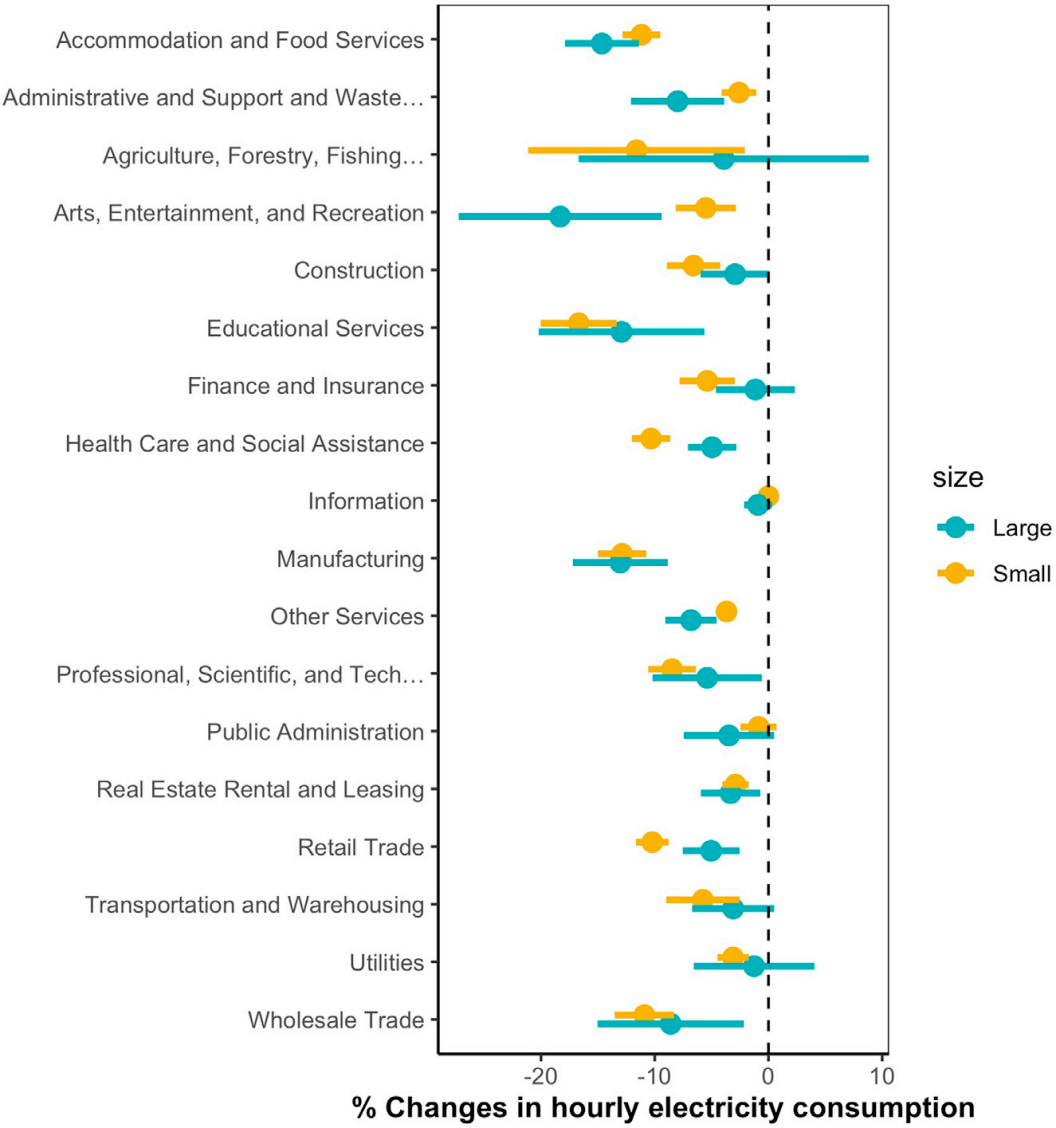
Percentage change in daily averaged hourly commercial electricity consumption because of the implementation of the school-closure/restricted business operation mandate by business size in Arizona The colored dots represent the percentage of changes in hourly electricity consumption, which are obtained from running the RDiT specification separately. The colored horizontal bars with corresponding colors represent the 95% confidence intervals of the estimations. We have dropped the mining sector from the figure due to insufficient observations.

Third, almost every industry bears a sizable drop in electricity consumption from the COVID-19 mandates, but those impacts vary across industry sizes, with small businesses tending to have a more pronounced decline in electricity consumption. The effects by the size of businesses within each industry sector in Arizona are plotted in Figure 6. In general, the majority of small industries (13 out of 18 industries) experienced larger declines in electricity consumption than medium- and large-sized industries. We find similar trends in Illinois (see Table S9). Especially for those industries with a higher percentage of employees in small businesses, namely, agriculture, forestry, fishing and hunting (83%), construction (82%), professional, scientific, and technical service (60%), wholesale trade (58%), educational services (45%), a larger decrease in the electricity consumption was observed for small-sized industries. It could pose a significant negative impact on the economy and job market because of their higher percentage of employees getting impacted, or their inability to maintain or reopen in the future (Dua et al., 2020). For a detailed breakdown of employment of small businesses, please see Table S6. Our results imply that small businesses in agriculture, forestry, fishing and hunting, retail and wholesale trade, health care and social assistance, construction, education services industries are more likely to experience negative economic impacts and thus the government should give priority and pay more attention to these types of businesses when releasing emergency subsidy packages. We highlight the need for focusing on small businesses because of their low financial resilience (Dua et al., 2020), meaning without proper policy incentives and support, they may never reopen, or recovery will take longer than larger companies.

We conducted the RDiT regression for each commercial consumer separately to explore the heterogeneous effects across individual commercial consumers' electricity consumption patterns changes resulting from mitigation measures. Figure S10 Panels B and Panel D indicate that the mitigation measures cause different treatment effects for each commercial user.

## Discussion

The COVID-19 mandates exacerbate the energy insecurity among low-income and ethnic minority groups based on the heterogeneous impact on electricity consumption. This paper reveals the disruption of economic and social activities caused by COVID-19 through changes in electricity consumption patterns. This disruption further reflects many electricity infrastructure issues that need more attention, such as the lower energy efficiency of homes occupied by the low-income and ethnic minority groups. The findings of this paper can serve as an essential piece for shaping future mandates and lockdown policies, which address the immediate impact following the first weeks of pandemics and natural disasters. In addition, we are also aware that our results are applicable for policies targeted to a negative shock in the short term at the scale of weeks. There is a consideration that people may adapt over the longer term by either adjusting their behaviors or finding alternative channels to deal with the shock. However, despite the fact that the initial policy reactions may not be appropriate for the longer term, our results will still provide some unique insights to relieve people's energy burden, especially for low-income communities. Our results are robust to a set of robustness checks, including a modified difference-in-difference (DID) analysis, a local two-step event study, and a pseudo RD check (see STAR Methods).

Our results directly show how COVID-19 mitigation measures increase energy insecurity and change the life pattern of the low-income and ethnic minority populations in the residential sector. A household's ability to meet their energy demand impacts their health, work productivity, and educational development (Jessel et al., 2019; Kumar, 2020; Vera and Langlois, 2007), and is shown in their changes in energy consumption pattern. Existing studies have found that people with lower socioeconomic status (Harrison and Popke, 2011), such as lower-income (Jacobs et al., 2014) and minority ethnicity (Adamkiewicz et al., 2014; Hernández et al., 2016; O'Neill et al., 2005), are associated with homes that are of lower-quality and less energy efficiency (Reames, 2016b; Jing Liang et al., 2020; Schill et al., 1998), and difficulty obtaining an adequate amount of energy to maintain a healthy indoor environment (Bednar et al., 2017). Meanwhile, we are fully aware that electricity is only one type of energy usage in the residential sector. The increase in electricity consumption in the residential sector was also echoed in water and gas usage (Almshekhs et al., 2021; Menneer et al., 2021). Thus, the implication of electricity consumption can serve as a part of larger energy dialogue. Our results can help policymakers to better quantify such negative impacts on energy insecurity among those under-represented groups during a pandemic. We also examine energy equality in the context of load profiles so that our findings can inform utilities about the energy insecurity impacts of demand-side management practices. The large increase in electricity consumption from 3 pm to 7 pm implies to the policymakers that a TOU rate that has peak hours with higher electricity prices during these hours might cause an even larger energy burden for vulnerable residential households and put more people at risk for falling behind on energy bills.

Beyond the residential sector, our results indirectly show how COVID-19 mitigation measures do not impose equal effects across business sectors. Impacts vary across industry sectors, where in-person retail and services, tend to receive a larger impact as illustrated by a larger decline in electricity consumption (Bartik et al., 2020). In addition, smaller businesses are more likely to have lower-wage workers, which leads to employment losses disproportionately concentrated in workers with a lower wage if small businesses were mostly impacted by the COVID-19 mandates (Cajner et al., 2020; Chetty et al. 2020). These heterogeneities in business sensitivities to the COVID-19 mandates introduce severe problems in the short-run and potentially irreversible damage in the long run due to hundreds of thousands of small businesses closing (Bartik et al., 2020; Hamilton, 2020). In turn, these closures could perpetuate economic and racial inequality due to disparities in access to capital resources (Fairlie and Robb, 2010) between minority and non-minority-owned businesses and the importance of small businesses to job creation for minority groups (Fairlie, 2020a). Our results show that policymakers can potentially use changes in electricity consumption patterns to quickly identify the most impacted small businesses during such a crisis so that the government can deliver the most needed financial relief packages.

Our findings reveal the energy insecurity and fragility of both low-income and ethnic minority populations as well as many small businesses due to the current pandemic. It highlights the importance of well-designed mandates and lockdown policies with a focus on vulnerable groups. Energy insecurity is correlated with low-income groups. However, compared with income inequities, energy insecurity has a more direct correlation with household well-being (Joyeux and Ripple, 2007). Therefore, energy insecurity should be considered a complementary measure for living standards, in addition to income (Joyeux and Ripple, 2007; Román-Collado and Colinet, 2018). Our results allow policymakers to provide precise mitigation measures that prioritize support and relief to vulnerable populations and small businesses to reduce the impacts on wellbeing and business operations (Graff and Carley, 2020; Liddell and Morris, 2010). When COVID-19 mitigation mandates are relaxed, evidence shows that unemployment will still be problematic and can cause broader racial and income inequality (Fairlie, 2020a; Goldman Sachs, 2020; Hamilton, 2020). Therefore, in addition to providing current unemployment insurance with extended benefits, subsidies on energy consumption are also an option to sustain residential wellbeing and small business operations.

Looking forward, work-from-home could become a new norm even in the post-COVID-19 era, where companies are seeking opportunities to re-allocate employee resources by encouraging remote work (Bloom, 2020; Gurchiek and Gurchiek, 2020; Philippa Fogarty et al., 2020; PricewaterhouseCoopers, 2020). Companies, such as Infosys, Nationwide Insurance, Shopify, Siemens, Slack, Square, Twitter, Upwork, and Zillow, are transitioning part or all of their employees to remote work permanently (FlexJobs, 2020). This trend will cause a persistent energy burden on low-income and ethnic minority households. On the other hand, work-from-home increased residential electricity consumption during the hours when solar irradiance is abundant. As a result, subsidies on solar power generation for low-income households could be more effective in relieving energy insecurity. This is particularly true with the large periods of time people are confined to their homes during the peak solar radiation hours.

## Conclusion

This paper assesses the impact of the COVID-19 related mandates on consumers' electricity consumption behaviors and the energy insecurity in Arizona and Illinois. Using the individual-consumer-level high-frequency electricity data and an RDiT approach, we investigate the hypothesis that electricity usage increase in the residential sector because of the COVID-19 measures will increase energy insecurity among low-income and ethnic minority groups. Our results indicate that the short-term impact of the COVID-19 mandates on low-income and ethnic-minority populations is greater than high-income and white populations in terms of residential electricity consumption increase. In the commercial sector, we find larger negative impacts for small businesses in certain industry sectors. Our ability to empirically identify and quantify energy insecurity because of COVID-19 mandates helps policymakers design more effective and equitable policies for energy poverty assistance.

### Limitations of the study

Our work demonstrates strong evidence that heterogeneous impacts of electricity consumption from COVID-19 mandates have manifested themselves in both the residential and commercial sectors. Nevertheless, several limitations are worth noting. First, the mandates do not provide a sharp discontinuity due to some consumers' panic during the pre-mandate period. However, we are confident that our results are robust due to the nature of the mandates in this paper. These mandates, such as school and business closure, will induce significant household behavior changes before and after the enforcement dates. Second, the RDiT approach is only able to identify the short-term shock of the COVID-19 mandates. Although we are not able to identify the long-term effects of the COVID-19 mandates, this work is a good first step in quantifying how COVID-19 impacted different demographic groups in the residential and different businesses in the commercial sector. Third, because of data limitations, we are only able to use demographic information at the zip-code level in Illinois. In future work, we recommend a high-resolution dataset to further investigate the energy usage discrepancies between households in this region. Fourth, our paper treats intra-group consumers homogeneously, and does not consider intra-group differences. For example, low-income groups might have specialized electricity rates (e.g., energy subsidy program), or small businesses may have different contracts relative to the larger companies.

## STAR★Methods

### Key resources table

**Table:** 

REAGENT or RESOURCE	SOURCE	IDENTIFIER
**Deposited data**		

Hourly electricity usage data in Illinois	ComEd	https://www.comed.com/SmartEnergy/InnovationTechnology/pages/anonymousdataservice.aspx
Hourly electricity usage data in Arizona	SPR	From SPR utility company directly
Social demographic data in Illinois	US Census	https://data.census.gov/
Hourly meteorological data	the National Oceanic and Atmospheric Administration (NOAA) Local Climatological Database	https://www.ncdc.noaa.gov/cdo-web/datatools/lcdE
Electricity price in Arizona	SPR Rate Book 2019	From SPR utility company directly
Individual-consumer-level demographic data in Arizona	2017 Residential Equipment and Technology Survey	From SPR utility company directly

**Software and algorithms**		

STATA 14	This study	https://www.stata.com/stata14/
RStudio Version 1.4.1103	This study	https://cran.r-project.org/
Python 3.8.8	This study	https://www.python.org/downloads/release/python-388/

### Resource availability

#### Lead contact

Further information and requests for resources and reagents should be directed to and will be fulfilled by the lead contact, Yueming (Lucy) Qiu (yqiu16@umd.edu)

#### Materials availability

This study did not generate new unique reagents.

### Method details

#### Data

Hourly electricity consumption data is provided by two electric services companies: the Salt River Project (SRP) of Arizona and the Commonwealth Edison (ComEd) of Illinois. Figure S1 illustrates the service territory maps for these two companies. SRP is one of the two primary electric utilities in Arizona, serving the Phoenix metropolitan area. ComEd is the sole provider in Chicago and Northern Illinois area. The Arizona data includes 7,004 residential (almost evenly split between TOU and non-TOU consumers) and 23,117 commercial users from January 1, 2019, to April 30, 2020. The Illinois data includes 40,771 residential accounts and 40,757 commercial accounts for the entire March 2020 (descriptive statistics in Tables S1–S5). The ComEd follows the same consumers for just one month, which limits the availability for panel data analysis. Thus, we adopted a one-month window for Illinois. Users of both datasets have their associated geographic area at the five-digit zip code level, which enables us to match meteorological and mobility variables at the same level through the spatial analysis tool QGIS.

Individual-consumer-level demographic data for the residential accounts in Arizona is compiled from a 2017 Residential Equipment and Technology Survey (response rate: 19%) conducted by SRP, where residential electric users are asked to provide detailed information in terms of the household income, socio-demographics, building conditions, and advanced technologies adopted. While we acknowledge that a survey with a response rate of 19% may not be representative of the full population, we find that there is a good distribution of residents across income, age, and ethnic groups. The RET survey data can be linked to the individual consumers in our smart meter dataset in Arizona. Demographic data for Illinois is collected from the American Community Survey of the United States Census Bureau. For Illinois data, we use QGIS to merge demographic information on income, race, and employment at the census ZIP Code Tabulation Areas (ZCTAs) level to each account. Although not as ideal as individual level data, zip-code level data can be used to evaluate correlations between social-demographics and electricity consumption (Cicala, 2021), but we note that they cannot be used to make causal statements. Commercial accounts in the Arizona dataset are provided with their six-digit code in the North American Industry Classification System (NAICS), which allows us to further break them into different industries based on their potential impacts from the COVID-19. Commercial accounts in Illinois are provided with information on their number of employees.

We obtained the hourly meteorological data from the National Oceanic and Atmospheric Administration (NOAA) Local Climatological Database (National Centers for Environmental, 2019). Additionally, we use mobility data to explore the mechanism that drives the change in electricity consumption patterns. Thus, we adopted mobility data from SafeGraph, where the hourly mobility information based on cellphone location data is available. Thus, we are able to calculate the hourly percentage of mobile devices staying at home from this dataset.

#### Mitigation measures

This analysis covers two types of mitigation measures that Arizona and Illinois state-level and city governments take during the COVID-19 pandemic: the state-wide school closure mandates, and constrained business operation hours.

In Arizona, Gov. Doug Ducey closed K-12 public and private schools on Monday, March 16, 2020 (Office of the Arizona Governor, 2020). Phoenix Mayor Kate Gallego declared a state of emergency for the city of Phoenix, at 8 pm on Tuesday, March 17^th^, forcing the closure of bars and moving restaurants to takeout, delivery, and drive-thru-only. Along with these efforts, other cities in the Phoenix-Mesa-Scottsdale metropolitan area (such as the city of Avondale, Tempe, and Scottsdale) also enforced similar mandates to reduce business operation hours on Monday, March 16^th^. Despite the State of Arizona issuing a state-wide executive order to limit business operations starting on March 20^th^ State of Arizona, 2020a, these local governments took action earlier. Starting at 5 pm on March 31^st^, 2020, the entire state followed the stay-at-home order State of Arizona, 2020b. Since these cities highly correspond to the service territory of the SRP, we adopted March 16^th^ as the threshold of the school closure and reduced business operations for analyses. In Illinois, the school was mandated to close on March 17^th^. Four days later, the entire state entered the stay-at-home order phase with business closed at the same time, which was 5 pm on March 21^st^ (State of Illinois, 2020a; State of Illinois, 2020b). Thus, the threshold of the school closure mandate for Illinois is March 17^th^. In both states, school-close orders were enforced in the morning so we use the same day as the threshold. Since the stay-at-home order happened later than the school closure mandate, we only focus on evaluating the school closure mandate because the behaviors changes were already induced by the school closure mandate prior to the start of the stay-at-home mandate. In our analyses of the school closure mandates, we dropped the dates after the start of the stay-at-home mandates to avoid confounding influence for the local specifications.

#### Empirical strategies

Our primary empirical strategy to estimate the treatment effect of COVID-19 related policies on electricity consumption is regression discontinuity in time (RDiT) (Lee and Lemieux, 2010), where the treatment starts on the day when the policy begins. In our case, the threshold is the school closure or restricting business date (*School*). We run the following equation:

(Equation 1) $$ln\left(EC_{iht\;}\right)=\;\alpha +\;\beta *\left(School_{t}\right)+\;\gamma *X_{iht}+f\left(Date_{t}\right)+\;\varepsilon_{iht}$$

where the logged hourly electricity consumption for consumer i at hour h on date t,$\;ln\left(EC_{iht\;}\right)\;$ is regressed on $\;\left(School_{t}\right)$. $\;\left(School_{t}\right)$ is equal to 1 if date *t* is after the school closure mandate took effect, respectively. $X_{iht}$ is a vector of covariates including indicators for the hour of the day, day of the week, the month of the year, and holiday dummy to control for electricity consumption patterns that vary by these time-dimensions, and also meteorological variables, including hourly temperature (in a restricted cubic spline format), precipitation (linear and quadratic format), air pressure, relative humidity, and wind speed. We understand that using month-of-year dummies might create sharp discontinuous jumps in residuals. Thus, we also ran models without the month of year dummies. The two pairs of results are very similar. Finally, we include a flexible *n*^*th*^ order polynomial $f\left(Date_{t}\right)$ to control for unobserved, time-varying factors. The $\varepsilon_{iht}\;$is the error term, and we clustered the standard errors at the account user level. The coefficient of interest, β, is the effect of the COVID-19 related policies on electricity consumption. β indicates the percentage change in electricity consumption resulting from COVID-19 mandates.

Existing RDiT literature suggests that RDiT designs normally have required observations far from the temporal threshold to capture the seasonal variation (Bento et al., 2014; Chen and Whalley, 2012; Davis, 2008; Hausman and Rapson, 2018). The biggest drivers of U.S. electricity consumption, outside of temperature, are the time of day, day of the week, and holidays (EIA, 2020b). Thus, a longer time window with controls for temperature and seasonal variation of electricity consumption can help prevent bias. A shorter time window will suffer from the correlations between the running variable and the latent variables, and the latter may result in discontinuous impacts on the potential outcome (Hausman and Rapson, 2018). For example, if the mandate was implemented on a Saturday, the potential outcome may evolve unevenly from the weekday to the weekend. Thus, adding sufficient control variables to capture seasonal variations are essential to understanding changes in electricity consumption patterns. We overcome data limitations using a refined strategy. We refine our RDiT model into a two-stage RDiT for Arizona analysis and fit the model with a group of covariates over a long 2-year winter-spring window.

(Equation 2) $$\;ln\left(EC_{iht\;}\right)=\;\alpha +\;\gamma *X_{iht}+\;\varepsilon_{iht}$$

We then save these residuals and regress the residuals on the policy change indicators only for the data of the year 2020. Because there is a gap between May 2019 to December 2019, which prevents us from running the regression discontinuity if we keep both 2019 and 2020 data:

(Equation 3) $$\;residual_{iht}\;=\delta +\;\beta *\;\left(School_{t}\right)+\;f\left(Date_{t}\right)+\theta_{iht}$$

For the Illinois dataset, we also use the two-stage approach but our window of analysis is limited to March 2020 because the availability of panel data for the ComEd data is only on monthly basis. However, despite the constraints of expanding the time dimension T, we improve precision by increasing the sample size, N. The number of account users in the Illinois case is almost twice as large as the Arizona case.

#### Local approach

we further take a local linear approach to validate the results from the polynomial approach. In this setting, a narrower bandwidth of 15 days before and after the policy effective dates (for Arizona) and 4 days (for Illinois) are adopted. We included different bandwidths for Arizona and Illinois because of the different periods between the school closures and stay-at-home order mandates (AZ -15 days, IL – 4 days). Thus, we use different bandwidths for the local linear regression to avoid confounding the impact of the stay-at-home order in the evaluation of the impact of the school-closure date. Additionally, we performed robustness checks with different bandwidths, presented in Table S11. We perform a similar two-step procedure as we did for the global polynomial approach, where the impacts of meteorological factors and seasonality are estimated, and residuals are saved by using the two-year window for Arizona and the entire window of March 2020 for Illinois. Then, we focus on a narrower window to perform the local linear estimation by regressing the residuals on the treatment without using a polynomial function of the time variables. Overall, results from the local linear approach in Tables S12 and S13 support our global polynomial approach.

#### Heterogeneity analysis

We take two different approaches to define the household income level due to the different data structures of these two states. First, residential consumers in Arizona are grouped into low, medium, and high income based on their household income per capita from the 2017 RET Survey. The 2017 survey was conducted by the utility company among their consumers. Thus, we merge the 2017 survey and the 2019 and 2020 electricity usage based on the unique ID number of each consumer. If consumers moved, they were dropped from this merge process. Thus, each consumer will have a set of social-demographic information. We adopted the division methodology provided by the Pew Research Center (Bennett et al., 2020), which is based on household income and household size (from one to five) in Phoenix-Mesa-Scottsdale metropolitan area. This is detailed further in Table S16. Additionally, we use a division methodology of the poverty line in the U.S. provided by the Department of Health & Human Services (Department of Health and Human Services, 2018) to construct a group of populations in poverty. We ran the same model on this group of consumers and find that the impacts of COVID-19 mandates on the population living with poverty are very similar to the low-income population. Figure S13 provides detailed results for the poverty population.

Second, in the state of Illinois, since the demographic data is aggregated at the ZCTAs level from the U.S. Census, we group our consumers as low, middle, and high income based on the level of the median wealth of the ZCTAs they belong to. We also adopted the division methodology provided by the Pew Research Center for the Chicago-Naperville-Elgin metropolitan area. When the median household income of the ZCTAs is below $57,000, residential consumers will be defined as a low-income group. ZCTAs with the median household income between $57,000 and $172,000 are middle-income groups, and those above $172,000 are defined as high-income groups.

For commercial users, we group them into small and large businesses based on their number of employees for Illinois data and the amount of pre-COVID electricity consumption for Arizona data. We adopt the definition of small business from the U.S. Small Business Administration in terms of the employment requirement, which indicates that small businesses do not exceed 500 employees (eCFR, 2020). The ComEd data comes with delivery classes as nonresidential consumers with the information on the number of employees, so we group these delivery classes into small and large businesses. The SPR data doesn't provide employment information, thus we adopted a method of calculating daily electricity consumption for each sector in the pre-COVID area and use the bottom 90% of the sample as small businesses and the rest of the 10% as the large business, similar to the categorization of small and large businesses from the Salesforce report (Salesforce, 2019).

#### Identification

RDiT design requires high-frequency data to be able to include flexible controls and to use the exact start dates of the treatment (Hausman and Rapson, 2018). Due to the mandatory nature of the treatment assignment, all consumers are moved to the treatment group following the COVID-19 mandates. Additionally, due to the characteristics of COVID-19 mandates, the effects from the mandates are immediate, a prerequisite for the RDiT design (Lee and Lemieux, 2010). The key driver of changes in electricity consumption behavior is based on the discontinuity in stay-at-home patterns on the implementation day of the mitigation measures for residential consumers, and the discontinuity in business operations for non-residential consumers. Thus, the identifying assumption of the RDiT is that there is a discontinuous change in the electricity consumption at the cut-off. These stay-at-home and business operations patterns are further supported by consumers' mobility patterns. Figures S5 and S6 display the mobility behavior changes throughout the mitigation measures in both states and clearly show the sharp discontinuity in daily mobility behavior on the first day of the mitigation measures.

We further validate our identification strategy through a pseudo RDiT strategy by adopting another timeframe, and making 2019 the pandemic year, and to see whether a placebo “pandemic” that happened in 2019 had any effect. If we can observe a similar discontinuous impact from the treatments in 2019, then our identification strategy is weak. Figures S7–S9 indicate that we do not observe an impact in 2019 for both residential and commercial users in both states, highlighting the robustness of our work.

#### Other robustness checks

We conduct robustness checks of the main results with two more alternative specifications including a modified difference-in-difference (DID) analysis and a local two-step event study, beyond the two robustness checks (pseudo RD and local RD estimates) described in the identification section. In the modified DID analysis, we treat Arizona consumers of 2019 as our control group and put these consumers into the same months in 2020. For Illinois, we put another group of consumers from March 2019 into March 2020 as the control group. We utilize the modified DID method to estimate the effects of the mitigation mandates on electricity consumption. Our modified DID uses the “pre-treatment” data in 2019 as the “control” group. There is no real “control” group in our study since all consumers are subject to the treatment of the mandates. Thus, we do not perform parallel trend testing like the traditional DID. In the local two-step event study, we adopted a similar strategy as our refined RDiT model. In the first stage, we fit the model with a group of covariates over a 2-year winter-spring window. We then save these residuals and regress the residuals on the policy change indicators within a timeframe of 15 days for Arizona and 4 days for Illinois. In Tables S7–S9 we performed DID estimates of our treatment effect. In Tables S26 and S27, we further conduct a local two-step event study. Overall, our estimates are highly robust and consistent across a variety of alternative specifications, strategies, and samples. Thus, our benchmark model is validated.

## Data Availability

• Data: Climate factors are obtained from U.S. Local Climatological Data (LCD) at https://data.nodc.noaa.gov/cgi-bin/iso?id=gov.noaa.ncdc:C00684. The high-frequency electricity data in Arizona are from the SRP and in Illinois are from ComEd. For SRP's data, as restricted by a non-disclosure agreement, they are available from the authors upon reasonable request and with permission from the SRP. Data from ComEd is available upon request from https://www.comed.com/SmartEnergy/InnovationTechnology/pages/anonymousdataservice.aspx. The mobility data is available upon request from the SafeGraph. All data and models are processed in Stata 14.0 and Python. The figures are produced in Python and R Studio. • Code: All custom code is available on GitHub from https://github.com/Jiehonglou/Inequitable-and-Heterogeneous-Impacts-on-Electricity-Consumption-from-COVID-19-Mitigation. • Any additional information required to reanalyze the data reported in this paper is available from the lead contact upon request.

## Supplemental information

Document S1. Figures S1–S13, Tables S1–S25, and Section S1
