# Supplementary material for: Inequitable and heterogeneous impacts on electricity consumption from COVID-19 mitigation measures
Source: iScience. 2021 Oct 7;24(11):103231. doi: 10.1016/j.isci.2021.103231 (PMC8494498; doi:10.1016/j.isci.2021.103231)
Supplement: Document S1. Figures S1–S13, Tables S1–S25, and Section S1 [file mmc1.pdf]

iScience, Volume *24*

Supplemental information

Inequitable and heterogeneous impacts on electricity consumption from COVID-19 mitigation measures

Jiehong Lou, Yueming (Lucy) Qiu, Arthur Lin Ku, Destenie Nock, and Bo Xing

This PDF file includes:

Figures. S1 to S13
Tables S1 to S28
Section S1 An analysis of state-wide stay-at-home orders

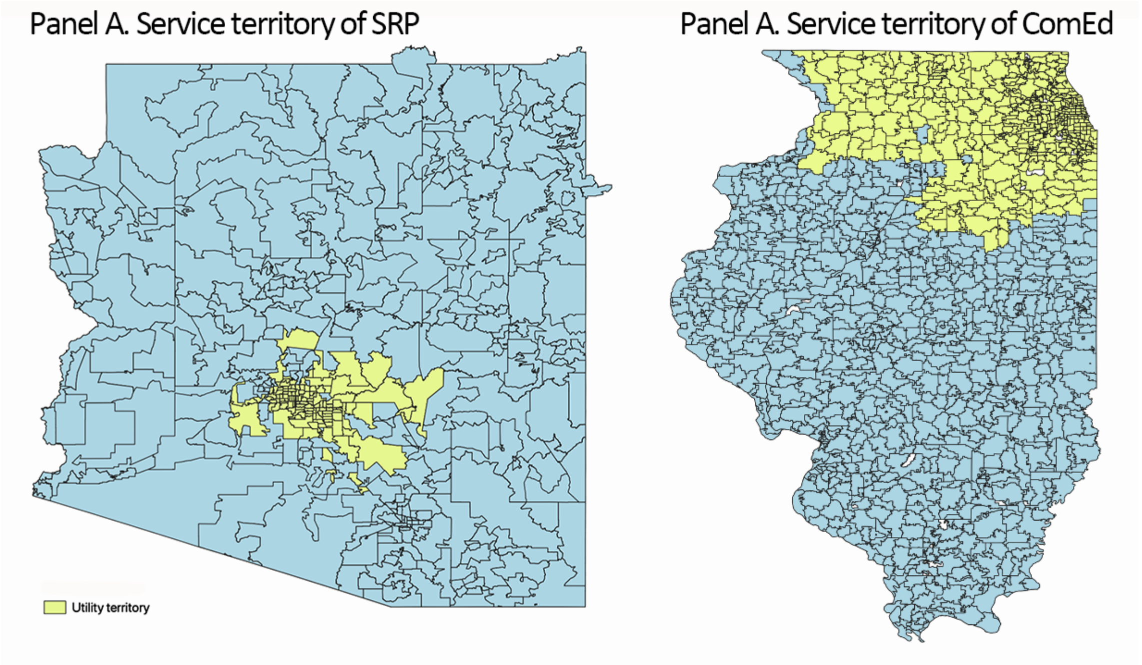

Figure S1. Utility service territory of SRP and ComEd. Panel A shows Arizona and Panel B shows Illinois. Related to STAR Methods.

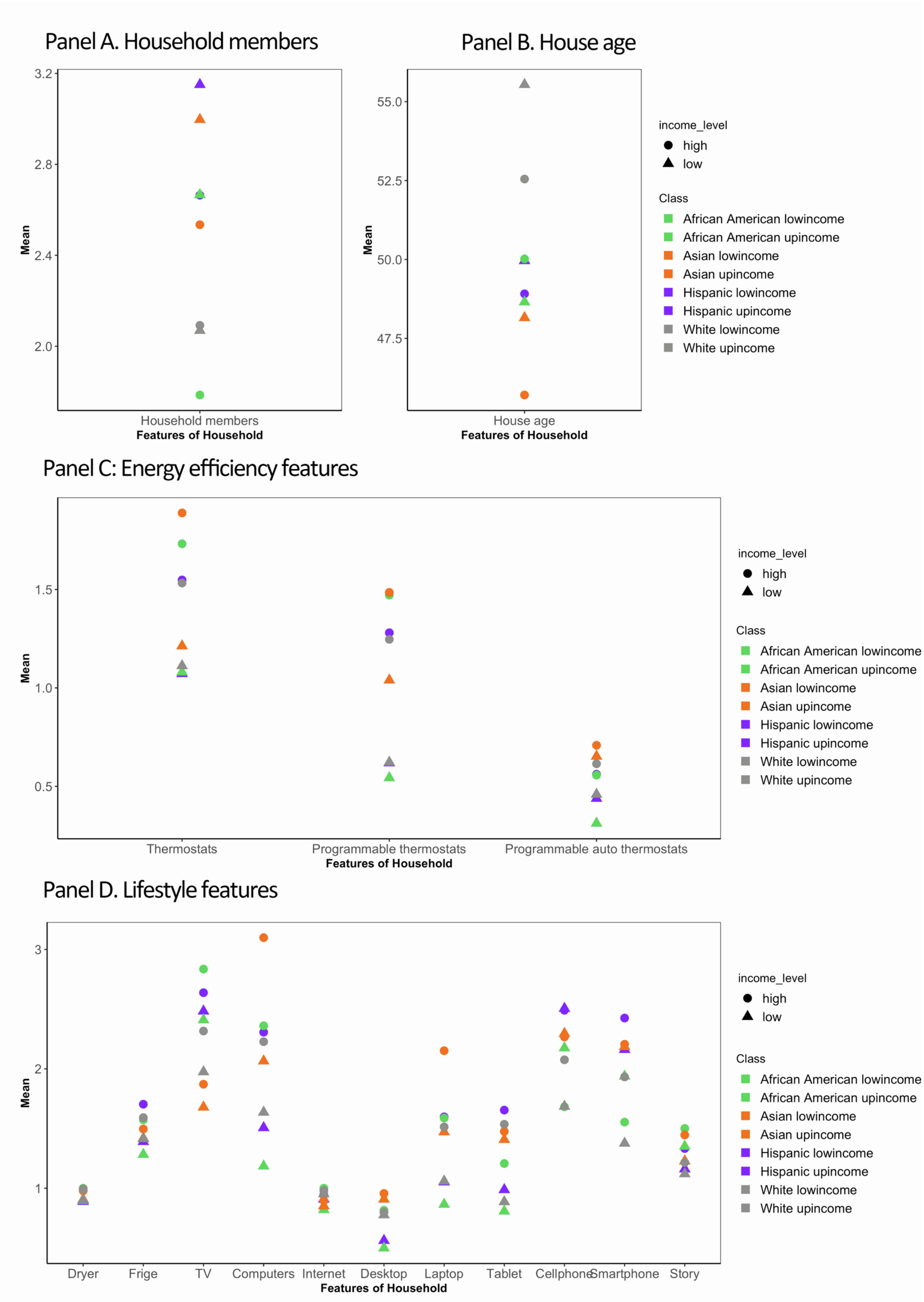

Figure S2. Arizona average number of features in a household by income and minority groups. Related to STAR Methods.

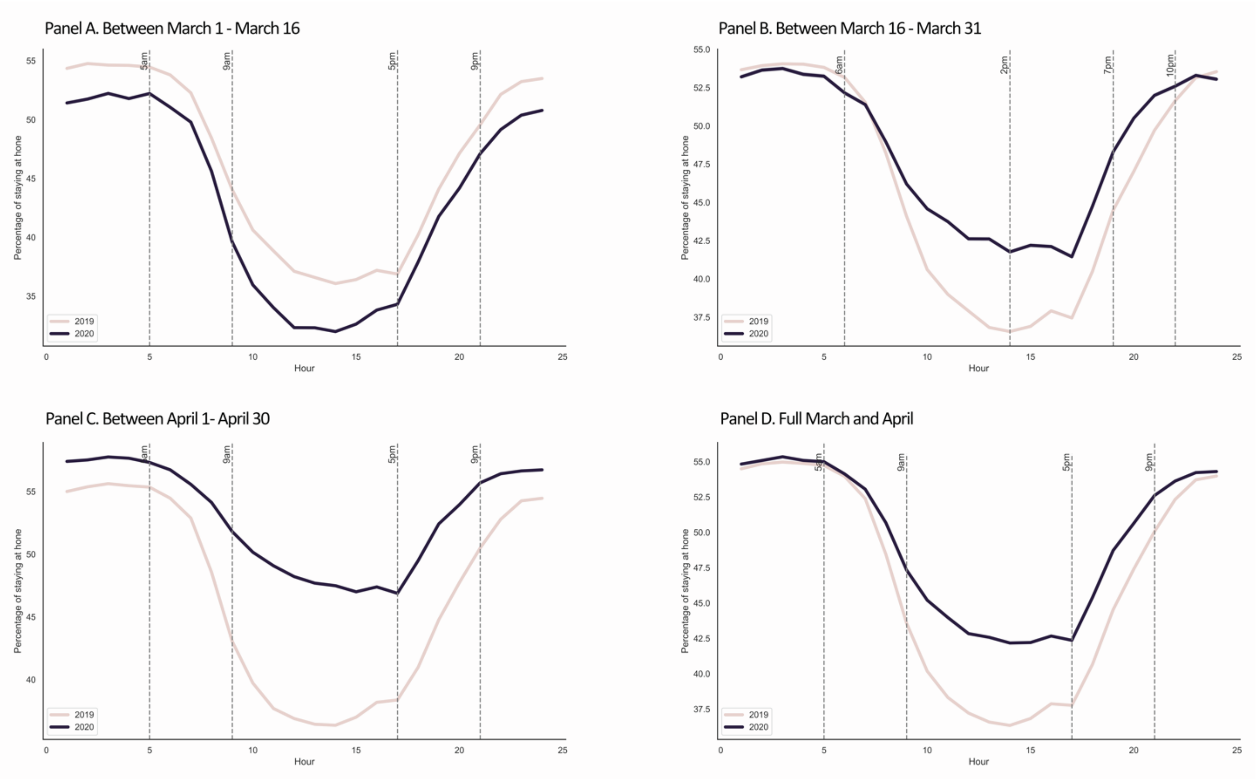

Figure S3. Arizona percentage of staying at home devices for the hour of the day. Related to STAR Methods. Panel A. Mobility percentage between March 1-March 15. Panel B. Mobility percentage between March 16-March 31. Panel C. Mobility percentage between April 1-April 30. Panel D. Mobility percentage for the entire March and April. Original devices (cell phones) data is from SafeGraph. (Sample size: 4170 block groups in AZ). Data source: SafeGraph mobility data

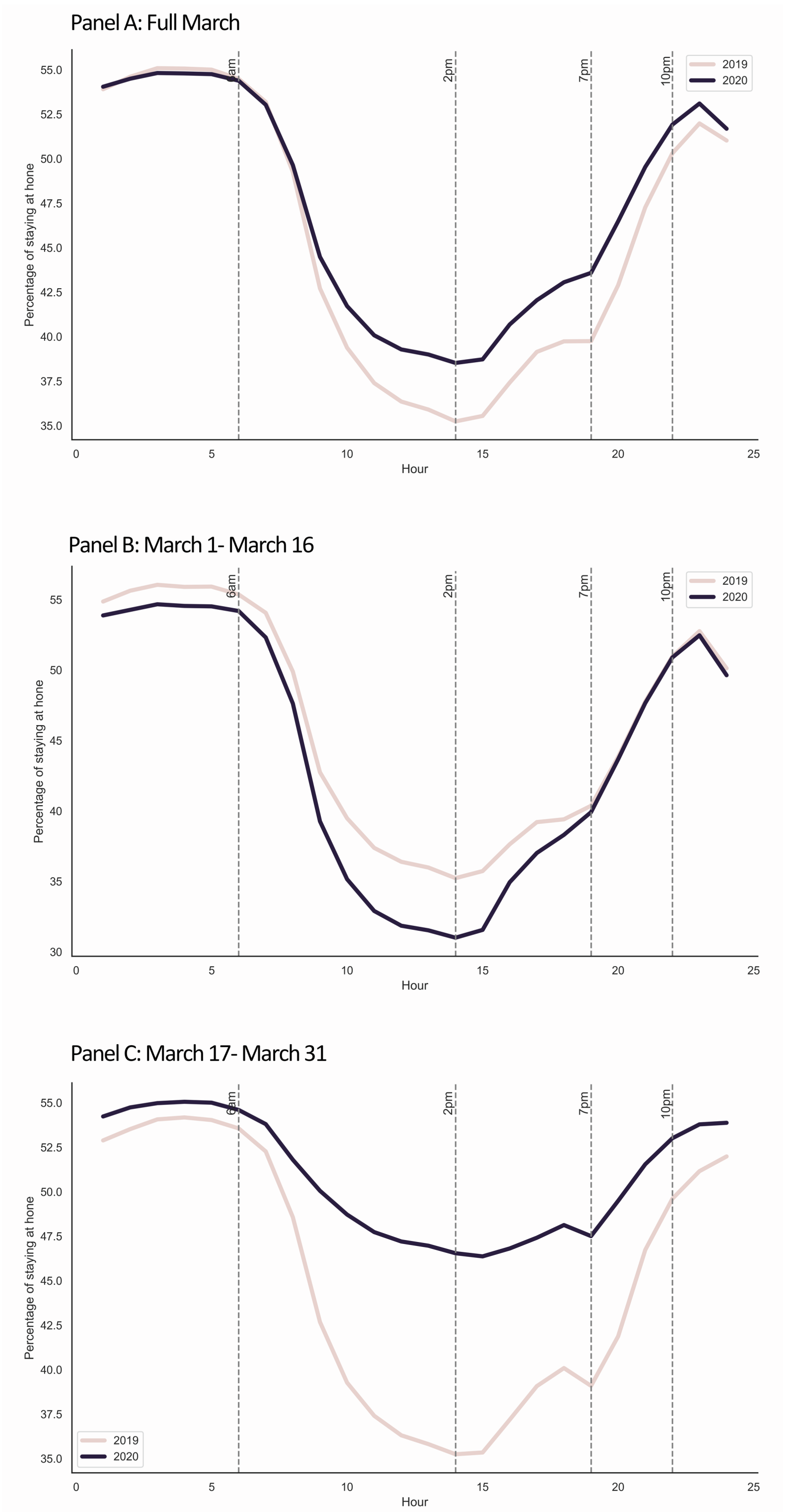

Figure S4. Illinois percentage of staying at home devices for the hour of the day. Related to STAR Methods. Panel A. Mobility percentage for the entire March. Panel B. Mobility percentage between March 1-March 16. Panel C. Mobility percentage between March 17-March 31.
Data source: SafeGraph mobility data

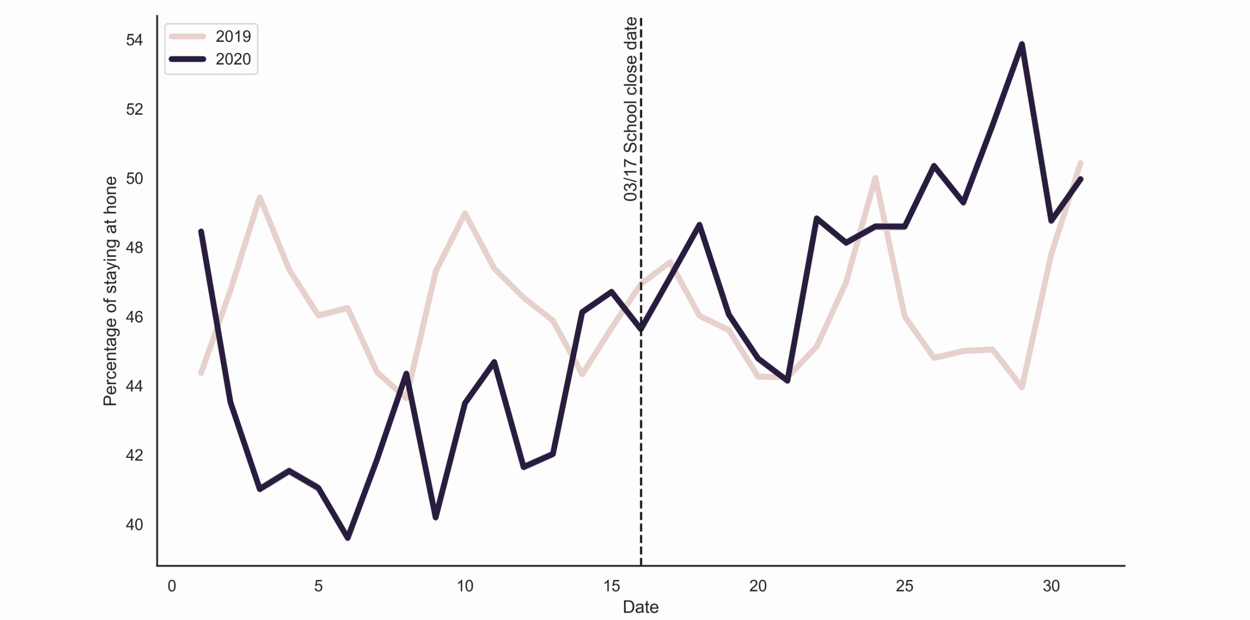

Figure S5. The Daily percentage of staying at home devices in Arizona in March. Related to STAR Methods. Data source: SafeGraph mobility data

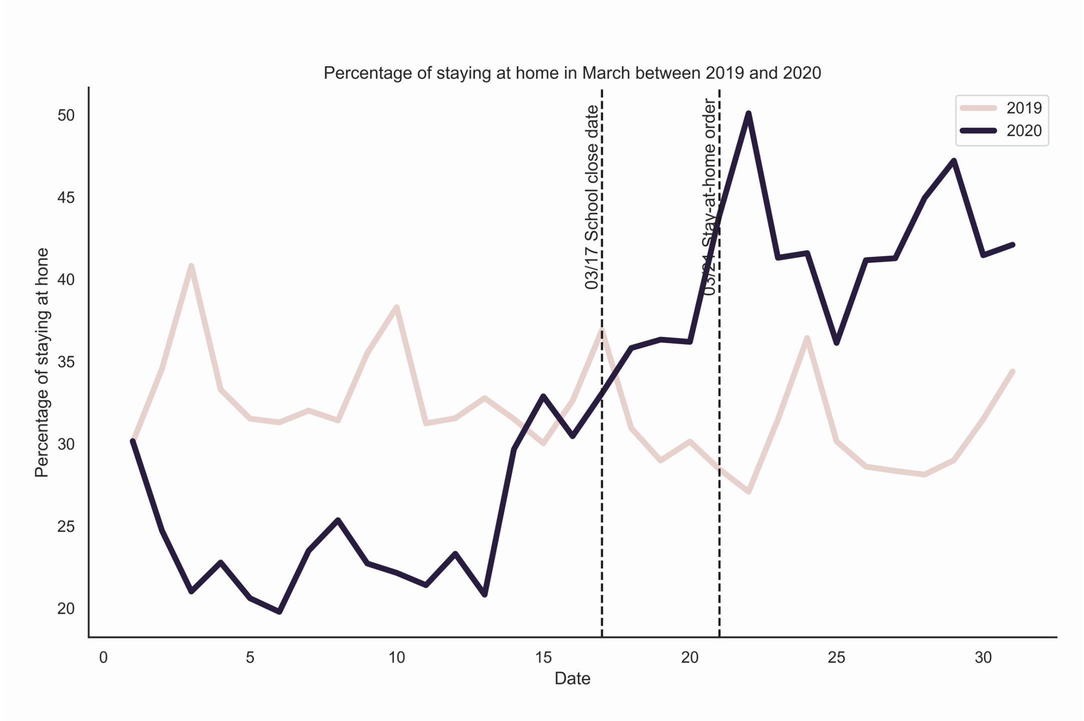

Figure S6. The Daily percentage of staying at home devices in Illinois in March. Related to STAR Methods.
Data source: SafeGraph mobility data

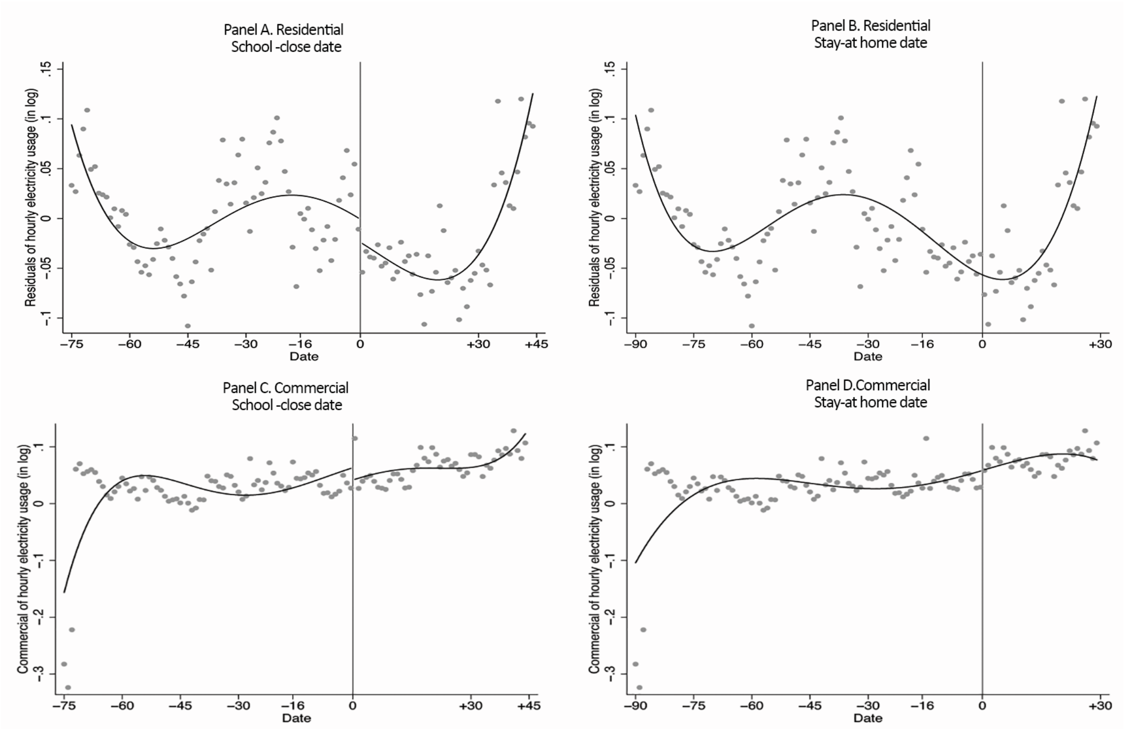

Figure S7. Residential and commercial daily averaged hourly electricity consumption percentage change in Arizona in 2019. Related to STAR Methods. The black circles are daily averaged hourly residuals of log electricity consumption (after controlling for covariates, such as weather) for Arizona. The consumption is averaged across all hours of the day.

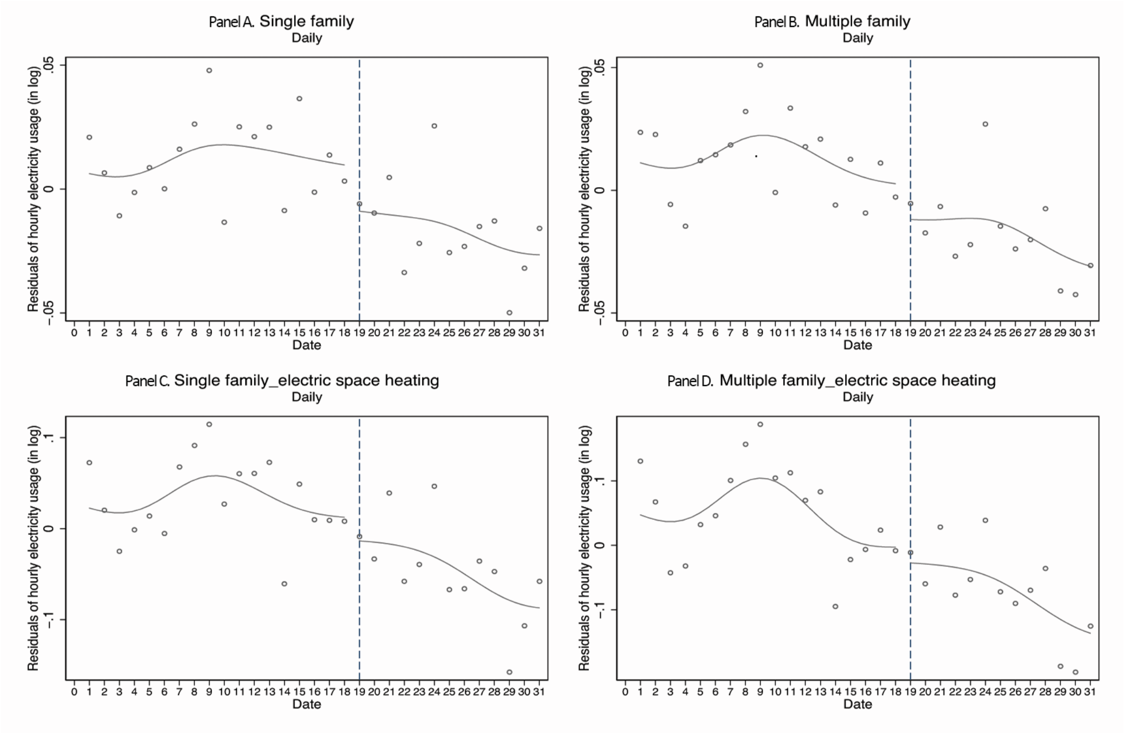

Figure S8. Residential daily averaged hourly electricity consumption percentage change in Illinois in 2019 for the school close policy. Related to STAR Methods.The black circles are daily averaged hourly residuals of log electricity consumption (after controlling for covariates, such as weather) for Illinois in March. Multiple family indicates multi-family home.

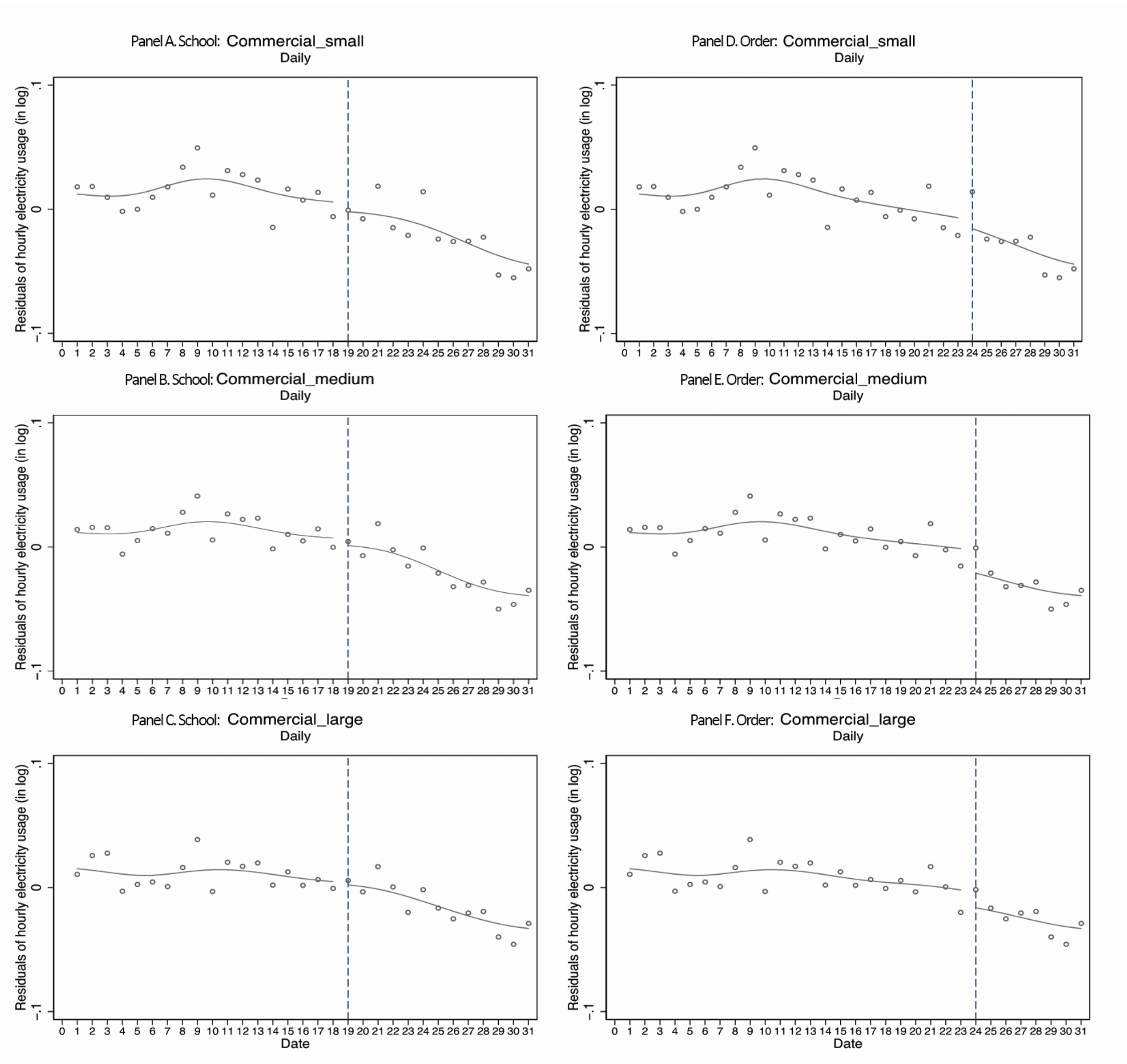

Figure S9. Commercial daily averaged hourly electricity consumption percentage change in Illinois in 2019, for school closure order policy. Related to STAR Methods. Black circles represent daily averaged hourly residuals of log electricity consumption (after controlling for covariates, such as weather) for Illinois in March.

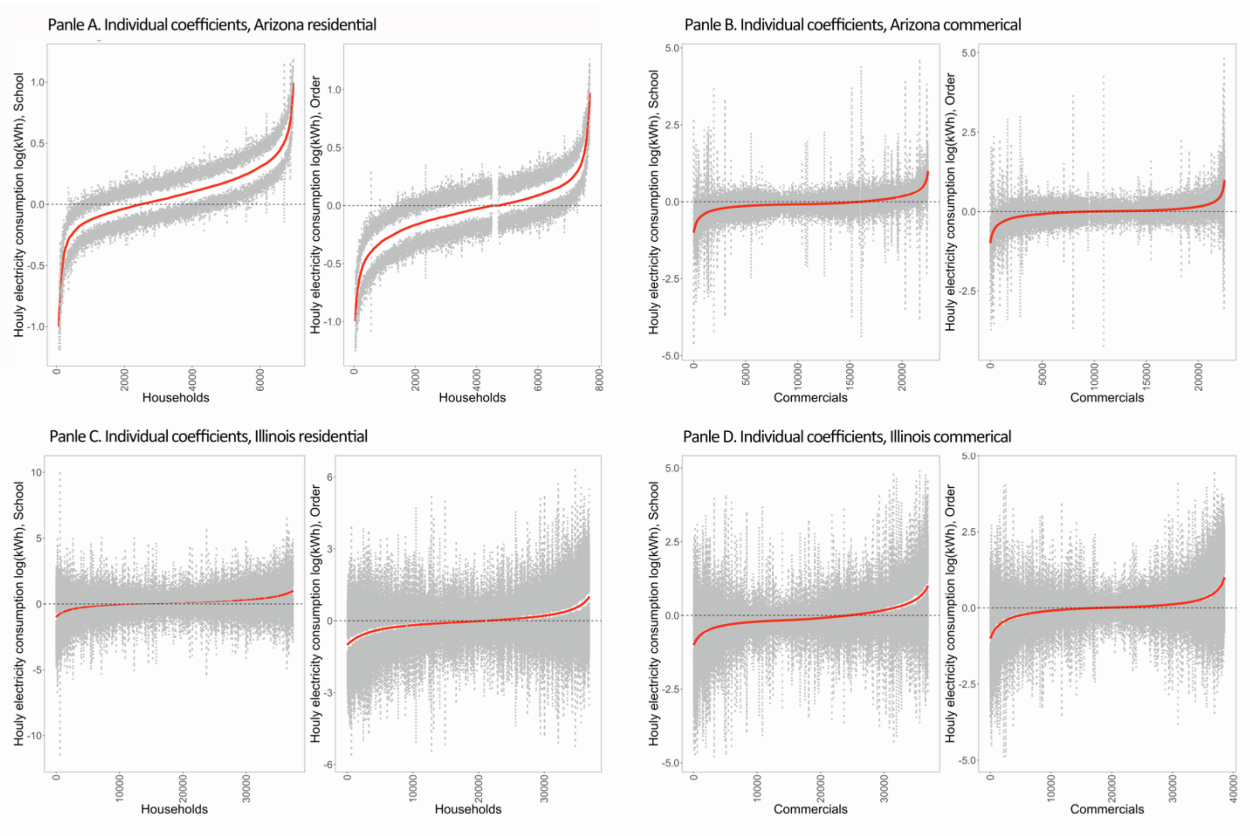

Figure S10. The individual coefficient of the effect of mitigation policies on residential and commercial hourly electricity consumption in Arizona and Illinois. Related to STAR Methods. The red line plots the coefficients of individual effect ranking from the smallest to the largest. Grey dotted lines represent the 95% confidence intervals.

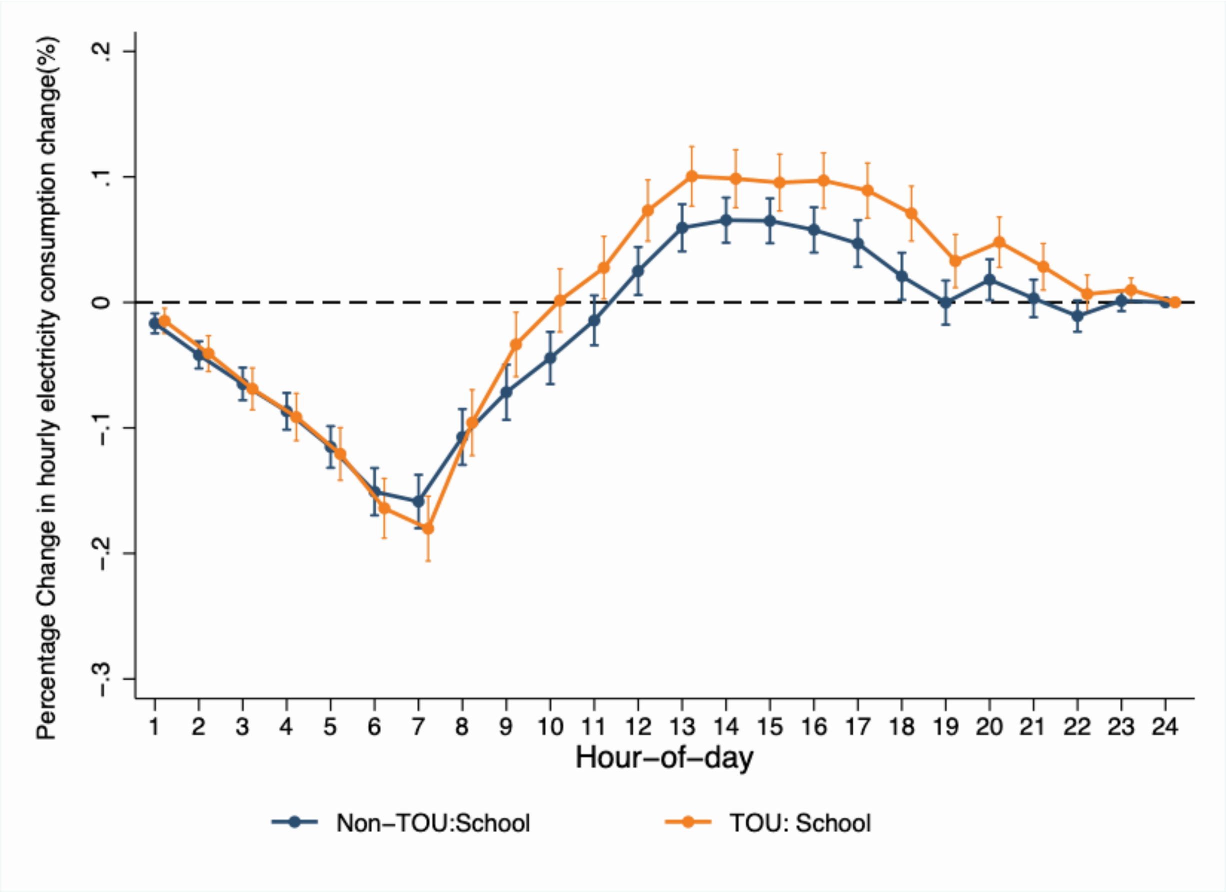

Figure S11. Percentage change in daily averaged hourly residential electricity consumption due to the implementation of the school closure/restricted business operation (yellow) mandates between TOU and non-TOU residential consumers. Related to STAR Methods. The colored dots represent the percentage of changes in hourly electricity consumption, which are obtained from running the RDiT specification separately. The colored horizontal bars represent the 95% confidence intervals of the estimations.

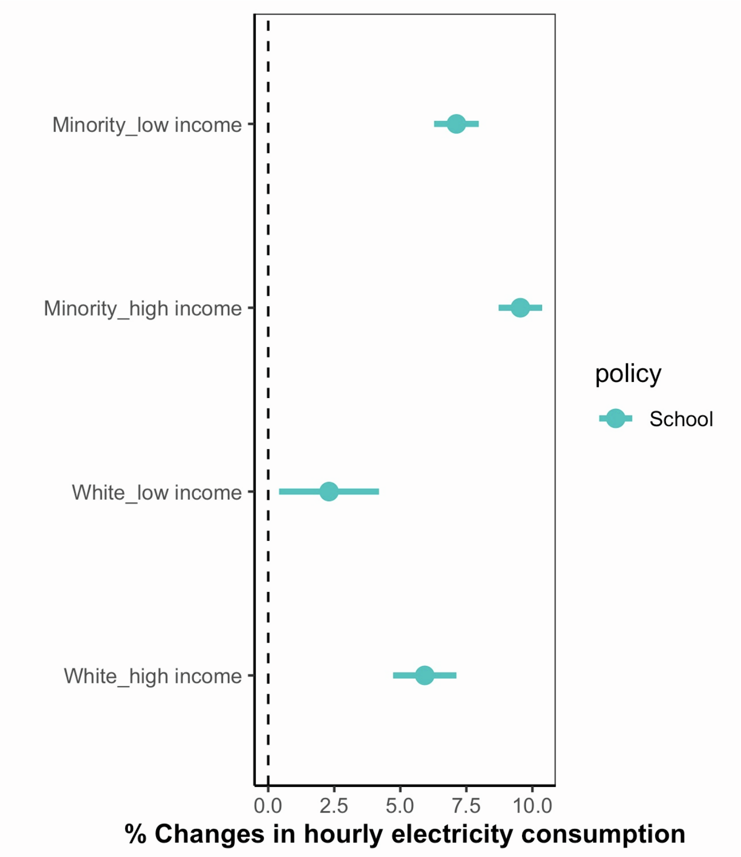

Figure S12. Percentage change in daily averaged hourly residential electricity consumption due to the implementation of the school closures/restricted business mandates by different demographic groups in Illinois. Related to Figure 2.The colored dots represent the percentage of changes in hourly electricity consumption, which are obtained from running the RDiT specification separately. Horizontal bars represent the 95% confidence intervals**.**

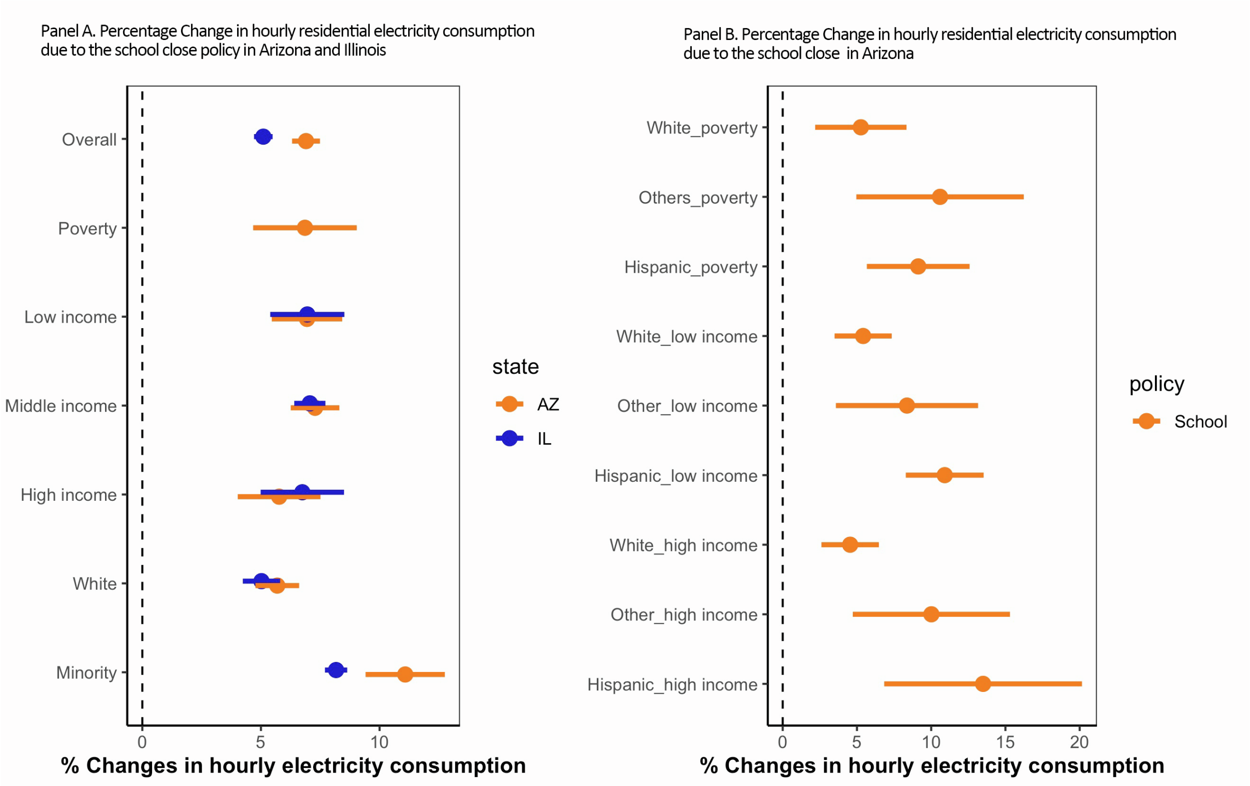

Figure S13. Percentage change in daily averaged hourly residential electricity consumption due to the implementation of the school closure/restricted business operation mandates by different demographic groups, adding poverty groups. Related to STAR Methods. The colored dots represent the percentage of changes in hourly electricity consumption, which are obtained from running the RDiT specification separately. The colored horizontal.

Table S1. Descriptive statistics of residential consumers in Arizona. Related to STAR Methods.

Variable	Obs	Mean	Std. Dev.	Min	Max	Unit
Hourly Electricity consumption	81,865,224	1.511	1.546	0.000	46.44	kWh
Temperature	81,865,224	70.328	17.475	27.000	115	Fahrenheit
Precipitation	81,865,224	0.001	0.008	0.000	0.59	Inch
Air pressure	81,865,224	28.577	0.192	27.97	29.26	inHg
Relative humidity	81,865,224	38.559	22.932	3.000	100	%
Wind speed	81,865,224	6.367	4.212	0.000	36	MPH(m/h)

Table S2. Descriptive statistics of commercial consumers in Arizona. Related to STAR Methods.

Variable	Obs	Mean	Std. Dev.	Min	Max	Unit
Hourly Electricity consumption	274,068,179	7.229	25.796	0.000	1096.356	kWh
Temperature	274,068,179	71.575	17.093	-1.000	115.000	Fahrenheit
Precipitation	274,068,179	0.001	0.009	0.000	0.590	Inch
Air pressure	274,068,179	28.631	0.201	23.540	29.240	inHg
Relative humidity	274,068,179	36.367	21.693	3.000	100.000	%
Wind speed	274,068,179	6.164	4.281	0.000	47.000	MPH (m/h)

Table S3. Descriptive statistics of residential consumers in Illinois. Related to STAR Methods.

	Variable	Observations	Mean	Std. Dev.	Min	Max	Unit
Single family – gas space heating	Hourly Electricity	15,031,032	0.833	0.799	0.000	55.404	kWh
	Temperature	15,031,032	37.918	10.142	-6.667	70.000	Fahrenheit
	Precipitation	15,031,032	0.002	0.016	0.000	0.850	Inch
	Air pressure	15,031,032	29.344	0.322	27.287	30.030	inHg
	Relative humidity	15,031,032	70.763	17.824	16.000	100.000	%
	Wind speed	15,031,032	10.144	5.738	0.000	41.333	MPH (m/h)
Multiple family – gas space heating	Hourly Electricity	8,038,920	0.393	0.499	0.000	22.524	kWh
	Temperature	8,038,920	38.070	9.732	-6.667	68.000	Fahrenheit
	Precipitation	8,038,920	0.002	0.014	0.000	0.755	Inch
	Air pressure	8,038,920	29.413	0.257	27.287	30.023	inHg
	Relative humidity	8,038,920	67.959	18.454	16.000	100.000	%
	Wind speed	8,038,920	10.704	5.360	0.000	41.333	MPH (m/h)
Single family – electric space heating	Hourly Electricity	17,704,224	2.811	2.531	0.000	112.404	kWh
	Temperature	17,704,224	37.567	10.470	-6.667	70.000	Fahrenheit
	Precipitation	17,704,224	0.003	0.016	0.000	0.850	Inch
	Air pressure	17,704,224	29.258	0.418	27.287	30.030	inHg
	Relative humidity	17,704,224	73.062	17.812	16.000	100.000	%
	Wind speed	17,704,224	9.699	5.935	0.000	41.333	MPH (m/h)
Multiple family – electric space heating	Hourly Electricity	20,457,768	1.343	1.509	0.000	45.053	kWh
	Temperature	20,457,768	38.102	9.909	-6.667	68.000	Fahrenheit
	Precipitation	20,457,768	0.003	0.015	0.000	0.850	Inch
	Air pressure	20,457,768	29.350	0.332	27.287	30.030	inHg
	Relative humidity	20,457,768	69.872	18.050	16.000	100.000	%
	Wind speed	20,457,768	10.292	5.564	0.000	41.333	MPH (m/h)

Table S4. Descriptive statistics of commercial consumers in Illinois. Related to STAR Methods.

	Variable	Observations	Mean	Std. Dev.	Min	Max	Unit
Commercial small (0-100)	Hourly Electricity	47,999,904	4.594	7.837	0.000	503.184	kWh
	Temperature	47,999,904	38.047	10.038	-6.667	68.000	Fahrenheit
	Precipitation	47,999,904	0.002	0.015	0.000	0.850	Inch
	Air pressure	47,999,904	29.360	0.312	27.287	30.030	inHg
	Relative humidity	47,999,904	69.919	18.004	16.000	100.000	%
	Wind speed	47,999,904	10.316	5.620	0.000	41.333	MPH (m/h)
Commercial medium (100-400)	Hourly Electricity	12,199,368	67.166	53.791	0.000	1442.010	kWh
	Temperature	12,199,368	38.057	10.129	-6.667	68.000	Fahrenheit
	Precipitation	12,199,368	0.003	0.015	0.000	0.755	Inch
	Air pressure	12,199,368	29.364	0.303	27.287	30.023	inHg
	Relative humidity	12,199,368	69.740	17.884	16.000	100.000	%
	Wind speed	12,199,368	10.341	5.627	0.000	41.333	MPH (m/h)
Commercial large (400-1000)	Hourly Electricity	1,839,912	245.609	146.403	0.000	1405.012	kWh
	Temperature	1,839,912	38.295	10.114	-4.000	68.000	Fahrenheit
	Precipitation	1,839,912	0.003	0.015	0.000	0.568	Inch
	Air pressure	1,839,912	29.378	0.284	27.287	30.023	inHg
	Relative humidity	1,839,912	68.976	17.978	16.000	100.000	%
	Wind speed	1,839,912	10.501	5.532	0.000	36.667	MPH (m/h)

Table S5. Descriptive statistics of commercial consumers by industry in Arizona. Related to STAR Methods.

	**# Of Accounts**	**Observations**	**Mean**	**Std. Dev.**	**Min**	**Max**
Accommodation and Food Services	900	5,211,072	16.06092	25.01061	0	491.004
Administrative and Support and Waste M..	769	4,464,939	2.47997	10.44703	0	894.156
Agriculture, Forestry, Fishing and Hunting	69	399,060	2.365366	5.780127	0	113.36
Arts, Entertainment, and Recreation	771	4,511,159	5.891981	15.16925	0	381.42
Construction	633	3,869,292	4.383631	16.76472	0	549.72
Educational Services	426	2,492,756	15.97043	31.03976	0	725.64
Finance and Insurance	360	2,082,105	8.257486	27.50771	0	739.98
Health Care and Social Assistance	1101	6,471,867	8.486003	27.36803	0	441.12
Information	2804	16,830,418	3.446641	18.78582	0	548.88
Manufacturing	979	5,720,179	23.24092	52.50048	0	797.22
Mining	7	46,270	94.97356	148.8498	0	825.3
Other Services	5363	31,648,081	1.949211	9.874669	0	562.6799
Professional, Scientific, and Technical	700	4,170,084	4.373296	15.43354	0	488.97
Public Administration	788	4,655,826	4.833688	17.57509	0	671.712
Real Estate Rental and Leasing	3074	17,992,917	4.515471	16.87926	0	540.1801
Retail Trade	1700	9,866,988	15.86572	39.19478	0	879.39
Transportation and Warehousing	559	3,238,882	9.757103	27.53869	0	513.36
Utilities	1526	9,334,753	1.048127	6.728027	0	255.84
Wholesale Trade	502	2,903,461	12.52632	33.43383	0	580.896

Table S6 Small business employment. Related to STAR Methods.

Sector	# of Sector Employees at Small Businesses (million)	% of Sector Employees at Small Businesses
Other Services	4.7	85%
Agriculture, Forestry, Fishing and Hunting	0.136	83%
Construction	5.4	82%
Real Estate Rental and Leasing	1.5	68%
Accommodation and Food Services	8.5	61%
Arts, Entertainment, and Recreation	1.4	60%
Professional, Scientific, and Technical Service	5.2	58%
Wholesale Trade	3.4	56%
Educational Services	1.6	45%
Health Care and Social Assistance	9	44%
Manufacturing	5	43%
Mining	0.244	42%
Retail Trade	5.5	35%
Transportation and Warehousing	1.7	35%
Administrative and Support and Waste Management and Remediation Services	3.8	32%
Finance and Insurance	1.9	30%
Information	0.984	28%
Utilities	0.111	17%

Data source: USAFacts: What is a “small” business and what sectors have the most of them? Updated on Sep.24, 2020.

Table S7 Difference-in-differences estimates of residential electricity usage (log). Related to STAR Methods.

	Arizona		Illinois
	School closure		School closure
**Covid-19 mandates**	0.059	***	0.046
	(0.005)		(0.001)
Weather-related variables	yes		yes
Month FE	yes		No
Day-of-week FE	yes		yes
Holiday FE	yes		No
Hourly FE	yes		yes
Account FE	yes		yes
Observations	39,881,232		30,283,001
Number of groups	14010		40,771
R-square	0.1002		0.0509

Notes: weather-related control variables include temperature (in a restricted cubic spline format), precipitation (linear and quadratic format), air pressure, relative humidity, and wind speed.
Standard errors, clustered by account id, are in parentheses.
*** Significant at the 1 percent level. ** Significant at the 5 percent level. * Significant at the 10 percent level.

Table S8 Difference-in-differences estimates of residential electricity usage (log). Related to STAR Methods.

	Arizona	
	School closure	
**Covid-19 mandates**	-0.052	***
	(0.002)	
Weather-related variables	yes	
Month FE	yes	
Day-of-week FE	yes	
Holiday FE	yes	
Hourly FE	yes	
Account FE	yes	
Observations	122,516,546	
Number of groups	23,117	
R-square	0.0205	

Notes: weather-related control variables include temperature (in a restricted cubic spline format), precipitation (linear and quadratic format), air pressure, relative humidity, and wind speed.
Standard errors, clustered by account id, are in parentheses.
*** Significant at the 1 percent level. ** Significant at the 5 percent level. * Significant at the 10 percent level.

Table S9 Difference-in-differences estimates of commercial electricity usage (log) in Illinois. . Related to STAR Methods.

	Commercial Small (0-100)		Commercial Medium (100-400)		Commercial Large (0-100)	
**School close (03/17/2020)**	-0.149	***	-0.166	***	-0.114	***
	(0.002)		(0.003)		(0.009)	
R-square	0.0778		0.18		0.1727	
Weather-related variables	yes		yes		yes	
Day-of-week FE	yes		yes		yes	
Hourly FE	yes		yes		yes	
Account FE	yes		yes		yes	

Table S10. Regression Discontinuity Estimates: Global Polynomial Results (Residential and Commercial Sector). Related to Figure 1 and Figure 4.

Polynomial order	3	4	5	6	7	8	BIC
**AZ_residential**	-0.005***	0.069***	0.053***	0.053***	0.032***	0.037***	0.053***
	(0.003)	(0.003)	(0.004)	(0.004)	(0.003)	(0.003)	(0.003)
Observations	19,998,526	19,998,526	19,998,526	19,998,526	19,998,526	19,998,526	19,998,526
Number of groups	7,004	7,004	7,004	7,004	7,004	7,004	7,004
R-square	0.00111	0.0021	0.0026	0.0027	0.0028	0.003	0.0021
**IL_residential**	0.044***	0.051***	0.021***	0.025***	-0.008***	0.008***	0.044***
	(0.002)	(0.002)	(0.002)	(0.002)	(0.002)	(0.002)	(0.002)
Observations	30,283,001	30,283,001	30,283,001	30,283,001	30,283,001	30,283,001	30,283,001
Number of groups	40,771	40,771	40,771	40,771	40,771	40,771	40,771
R-square	0.001	0.001	0.001	0.002	0.002	0.002	0.001
**AZ_commercial**	-0.064***	-0.040***	-0.050***	-0.040***	-0.038***	-0.045***	-0.050***
	(0.002)	(0.002)	(0.002)	(0.002)	(0.002)	(0.002)	(0.002)
Observations	37,564,057	37,564,057	37,564,057	37,564,057	37,564,057	37,564,057	37,564,057
Number of groups	14,271	14,271	14,271	14,271	14,271	14,271	14,271
R-square	0.001	0.001	0.002	0.002	0.002	0.002	0.002
**IL_commercial**	-0.076***	-0.068***	-0.043***	-0.037***	-0.045***	-0.068***	-0.068***
	(0.002)	(0.002)	(0.002)	(0.002)	(0.002)	(0.002)	(0.002)
Observations	30,114,636	30,114,636	30,114,636	30,114,636	30,114,636	30,114,636	30,114,636
Number of groups	40.757	40,757	40,757	40,757	40,757	40,757	40,757
R-square	0.001	0.001	0.001	0.001	0.001	0.001	0.001

Table S11. Regression discontinuity estimates of residential and commercial electricity usage (log) of the school closure mandate: local estimates of 15 days for Arizona and 4 days for Illinois. Related to Table 1.

Local bandwidths	**Full window**	**7 days**	**14 days**	**30 days**	**45 days**
**AZ_residential**	0.024***	0.012***	0.009***	0.019***	0.003***
	(0.003)	(0.004)	(0.003)	(0.003)	(0.003)
Observations	21,661,642	2,479,234	4,962,531	10,877,398	16,054,720
Number of groups	7,714	6,999	7,581	7,628	7,657
R-square	0.0007	0.001	0.0014	0.0021	0.0007
**AZ_commercial**	-0.059***	-0.010***	-0.025***	-0.054***	-0.069***
	(0.002)	(0.004)	(0.003)	(0.002)	(0.002)
Observations	37,564,057	4,636,216	8,972,989	18,881,272	27,867,807
Number of groups	14,271	14,080	14,092	14,160	14,190
R-square	0.001	0.001	0.002	0.002	0.002
	**Full window**	**16-4 days**			
**IL_residential**	0.068***	0.099***			
	(0.002)	(0.002)			
Observations	30,283,001	20,514,988			
Number of groups	40,771	40,771			
R-square	0.0009	0.0013			
**IL_commercial**	-0.078***	-0.048***			
	(0.001)	(0.002)			
Observations	30,114,636	20,402,246			
Number of groups	40.757	40,757			
R-square	0.001	0.001			

Note: 16-4 days means that the time window covers 16 days before, and 4 days after the school closure date.

Table S12. Regression discontinuity estimates of commercial electricity usage (log) of the school closure mandate: local estimates of 15 days for Arizona and 4 days for Illinois. Related to Table 1.

	Arizona		Illinois
	School closure		School closure
**Covid-19 mandates**	0.012	***	0.031
	(0.003)		(0.002)
Weather-related variables	yes		yes
Month FE	yes		No
Day-of-week FE	yes		yes
Holiday FE	yes		No
Hourly FE	yes		yes
Account FE	yes		yes
Observations	5,530,149		8,793,256
Number of groups	7,595		40,771
R-square	0.0015		0.0018

Notes: weather-related control variables include temperature (in a restricted cubic spline format), precipitation (linear and quadratic format), air pressure, relative humidity, and wind speed.
Standard errors, clustered by account id, are in parentheses.
*** Significant at the 1 percent level. ** Significant at the 5 percent level. * Significant at the 10 percent level.

Table S13. Regression discontinuity estimates of commercial electricity usage (log) of the stay-at-home mandate: local estimates of 15 days for Arizona and 4 days for Illinois. . Related to STAR Methods.

	Arizona		Illinois
	School closure		School closure
**Covid-19 mandates**	-0.023	***	-0.088
	(0.002)		(0.002)
Weather-related variables	yes		yes
Month FE	yes		No
Day-of-week FE	yes		yes
Holiday FE	yes		No
Hourly FE	yes		yes
Account FE	yes		yes
Observations	15,209,855		8,743,262
Number of groups	22,771		40,757
R-square	0.0015		0.0069

Notes: weather-related control variables include temperature (in a restricted cubic spline format), precipitation (linear and quadratic format), air pressure, relative humidity, and wind speed.
Standard errors, clustered by account id, are in parentheses.
*** Significant at the 1 percent level. ** Significant at the 5 percent level. * Significant at the 10 percent level.

Table S14 Regression discontinuity estimates of residential electricity usage (log) of the school closure mandate heating type: global results (daily) 4th polynomial for school closure mandate. Related to STAR Methods.

	House with gas heating		House with electric space heating		Residential whole	
**School close (03/16/2020)**	0.087	***	0.059	***	0.069	***
	(0.006)		(0.004)		(0.003)	
Weather-related variables	yes		yes		yes	
Month FE	yes		yes		yes	
Day-of-week FE	yes		yes		yes	
Holiday FE	yes		yes		yes	
Hourly FE	yes		yes		yes	
Account FE	yes		yes		yes	
Observations	5,209,256		14,317,768		19,998,526	
Number of groups	1,835		5,003		7,004	
R-square	0.0039		0.0018		0.0021	

Notes: weather-related control variables include temperature (in a restricted cubic spline format), precipitation (linear and quadratic format), air pressure, relative humidity, and wind speed.
Standard errors, clustered by account id, are in parentheses.
*** Significant at the 1 percent level. ** Significant at the 5 percent level. * Significant at the 10 percent level.

Table S15 Regression discontinuity estimates of commercial electricity usage (log) of the COVID-19 mandates by industry size: global results (daily) 4th polynomial for school close policy and 3rd polynomial for order policy. Related to STAR Methods.

	Commercial Small (0-100)		Commercial Medium (100-400)		Commercial Large (0-100)	
**School close (03/17/2020)**	-0.050	***	-0.077	***	-0.040	***
	(0.002)		(0.005)		(0.013)	
Weather-related variables	yes		yes		yes	
Day-of-week FE	yes		yes		yes	
Hourly FE	yes		yes		yes	
Account FE	yes		yes		yes	
Observations	17,046,324		4,445,028		715,768	
Number of groups	31,599		7,964		1,194	

Notes: weather-related control variables include temperature (in a restricted cubic spline format), precipitation (linear and quadratic format), air pressure, relative humidity, and wind speed.
Standard errors, clustered by account id, are in parentheses.
*** Significant at the 1 percent level. ** Significant at the 5 percent level. * Significant at the 10 percent level.

Table S16. Household income and the number of people in your household in Arizona. Related to STAR Methods.

Household number	Low	Middle	High
1	$27,000	$82,000	$83,000
2	$38,000	$116,000	$117,000
3	$47,000	$142,000	$143,000
4	$54,000	$164,000	$165,000
5	$61,000	$184,000	$185,000
6	$67,000	$201,000	$202,000
7	$72,000	$218,000	$219,000
8	$77,000	$233,000	$234,000

Data source: the Pew Research Center.

Table S17. Descriptive statistics of residential consumers in Arizona in terms of # of household and house age. Related to STAR Methods.

	Income and Minority Groups	Observations	Mean	Std. Dev.	Min	Max
**House age**	White low income	3,791,185	55.53969	13.11535	20	70
	White high income	4,865,848	52.54839	11.33761	20	70
	Hispanic low income	1,378,593	49.95948	11.72176	20	70
	Hispanic high income	405,407	48.91186	10.82895	20	70
	Others low income	702,525	48.55648	12.1395	20	70
	Others high income	540,553	48.13101	11.34987	20	70
**# of households occupants**	White low income	4,207,555	2.069235	1.079578	1	5
	White high income	5,332,731	2.092077	0.9154318	1	5
	Hispanic low income	1,469,974	3.15069	1.21151	1	5
	Hispanic high income	445,118	2.664302	1.334644	1	5
	Others low income	748,796	2.807153	1.16799	1	5
	Others high income	575,062	2.312081	1.263522	1	5

Table S18 Descriptive statistics of residential consumers in Arizona in terms of energy efficiency features. Related to STAR Methods.

Efficiency	**Income and Minority Groups**	**Observations**	**Mean**	**Std. Dev.**	**Min**	**Max**
Thermostats	White low income	4,110,588	1.11246	0.3647109	1	3
	White high income	5,326,947	1.532699	0.6559168	1	3
	Hispanic low income	1,423,703	1.072176	0.2735312	1	3
	Hispanic high income	445,118	1.548252	0.6109478	1	3
	Others low income	709,081	1.175082	0.4561173	1	3
	Others high income	569,472	1.68481	0.7567229	1	3
Programmable thermostats	White low income	4,064,900	0.6220379	0.6076346	0	3
	White high income	5,315,382	1.246559	0.8518381	0	3
	Hispanic low income	1,412,135	0.6189819	0.5335915	0	2
	Hispanic high income	445,118	1.280166	0.7972621	0	3
	Others low income	674,761	0.818847	0.729592	0	3
	Others high income	569,472	1.365914	0.9438115	0	3
Programmable thermostats with auto function	White low income	2,261,343	0.4594009	0.4983491	0	1
	White high income	4,295,433	0.6145923	0.4866915	0	1
	Hispanic low income	822,610	0.4385031	0.496204	0	1
	Hispanic high income	365,308	0.5615207	0.4962014	0	1
	Others low income	433,970	0.5277623	0.4992292	0	1
	Others high income	461,127	0.6642942	0.4722371	0	1

Table S19. Descriptive statistics of residential consumers in Arizona in terms of # of features of lifestyles. Related to STAR Methods.

Lifestype	Income and Minority Groups	Observations	Mean	Std. Dev.	Min	Max
Square feet	White low income	4,093,453	1511.141	534.1788	1000	4000
	White high income	5,309,790	2270.573	805.6642	1000	4000
	Hispanic low income	1,395,191	1561.015	584.582	1000	4000
	Hispanic high income	445,118	2314.394	860.1374	1000	4000
	Others low income	714,287	1669.351	735.9592	1000	4000
	Others high income	569,278	2386.942	943.6903	1000	4000
Story	White low income	3,995,691	1.120183	0.3507309	1	3
	White high income	5,240,972	1.216049	0.4269027	1	3
	Hispanic low income	1,389,625	1.161194	0.4104981	1	3
	Hispanic high income	428,344	1.332597	0.4711441	1	2
	Others low income	691,726	1.246661	0.5021536	1	3
	Others high income	563,880	1.383603	0.5452671	1	3
Fridge	White low income	4,173,238	1.41474	0.5980387	1	3
	White high income	5,298,226	1.591711	0.6802463	1	3
	Hispanic low income	1,435,850	1.390324	0.5775581	1	3
	Hispanic high income	445,118	1.703744	0.700617	1	3
	Others low income	743,204	1.446961	0.6092478	1	3
	Others high income	575,062	1.560833	0.7078624	1	3
Dryer	White low income	4,173,437	0.9008247	0.2988973	0	1
	White high income	5,326,948	0.9935578	0.0800041	0	1
	Hispanic low income	1,446,839	0.8886144	0.3146091	0	1
	Hispanic high income	445,118	0.987437	0.1113785	0	1
	Others low income	743,012	0.8834945	0.3208304	0	1
	Others high income	575,062	0.9798839	0.1403975	0	1
TV sets	White low income	4,178,831	1.97434	0.9283312	0	4
	White high income	5,321,357	2.316763	1.003567	0	4
	Hispanic low income	1,464,214	2.483331	0.9958745	0	4
	Hispanic high income	445,118	2.637894	0.9594287	1	4
	Others low income	743,012	2.162172	1.02962	0	4
	Others high income	575,062	2.234982	1.126922	0	4
Computer	White low income	4,001,696	1.636084	1.252845	0	9
	White high income	5,287,237	2.22749	1.338932	0	11
	Hispanic low income	1,390,153	1.506848	1.237439	0	7
	Hispanic high income	445,118	2.307002	1.359461	0	6
	Others low income	708,693	1.729863	1.36993	0	7
	Others high income	575,062	2.805557	1.620491	0	7
Desktop	White low income	3,080,486	0.7764574	0.8305673	0	6
	White high income	4,668,763	0.7988495	0.9768649	0	10
	Hispanic low income	1,109,297	0.562376	0.7368379	0	4
	Hispanic high income	371,279	0.8006055	0.8277094	0	3
	Others low income	588,004	0.7370477	0.9138462	0	5
	Others high income	535,158	0.9556897	1.050857	0	4
Laptop	White low income	3,080,486	1.059683	0.9592198	0	6
	White high income	4,668,763	1.514	1.027961	0	6
	Hispanic low income	1,109,297	1.051763	0.9241524	0	5
	Hispanic high income	371,279	1.598087	1.155813	0	6
	Others low income	588,004	1.162621	0.9857047	0	5
	Others high income	535,158	1.858429	1.105302	0	5
Tablet	White low income	3,949,833	0.8864955	1.067299	0	6
	White high income	5,219,020	1.535813	1.151473	0	8

	Hispanic low income	1,360,852	0.9875313	1.139219	0	5
	Hispanic high income	433,552	1.654447	1.387323	0	6
	Others low income	703,100	1.23086	1.254486	0	7
	Others high income	575,062	1.665773	1.600371	0	10
Cellphone	White low income	4,098,852	1.685601	0.9422966	0	5
	White high income	5,326,948	2.075896	0.8870139	0	10
	Hispanic low income	1,441,440	2.505943	1.176491	0	9
	Hispanic high income	439,335	2.491013	1.098803	1	5
	Others low income	726,044	2.203416	1.244886	0	6
	Others high income	575,062	2.106933	1.002317	0	5
Smartphone	White low income	3,702,295	1.376395	1.057308	0	5
	White high income	5,235,597	1.932339	0.9445983	0	10
	Hispanic low income	1,395,171	2.161665	1.34536	0	9
	Hispanic high income	439,335	2.425623	1.108264	1	5
	Others low income	651,820	1.988185	1.281875	0	5
	Others high income	563,882	2.048886	0.9877858	0	5
Internet	White low income	3,406,799	0.9530345	0.211565	0	1
	White high income	5,099,868	0.9807972	0.1372374	0	1
	Hispanic low income	1,121,605	0.9082092	0.2887305	0	1
	Hispanic high income	422,176	0.9452006	0.2275886	0	1
	Others low income	598,994	0.8757617	0.3298535	0	1
	Others high income	569,278	0.9299077	0.2553027	0	1

Table S20. Descriptive statistics of residential consumers in Arizona in terms of electricity consumption. Related to STAR Methods.

Variable	Income and Minority Groups	Obs	Mean	Std. Dev.	Min	Max
electricity consumption	Low_income	6,517,515	1.051228	1.103497	0	17.599
electricity consumption	Middle_income	16,063,677	1.148415	1.163751	0	23.1312
electricity consumption	High_income	6,444,481	1.392708	1.449742	0	40.84

Variable	Income and Minority Groups	Obs	Mean	Std. Dev.	Min	Max
electricity consumption	White_low_income	4,207,555	0.9967372	1.061486	0	16.25
electricity consumption	White_high_income	5,332,731	1.401708	1.441789	0	40.84
electricity consumption	Hispanic_low_income	1,469,974	1.153048	1.167132	0	14.0826
electricity consumption	Hispanic_high_income	445,118	1.428496	1.328708	0	23.8794
electricity consumption	Other_low_income	748,796	1.130466	1.164438	0	17.599
electricity consumption	Other_high_income	575,062	1.240659	1.543826	0	29.518

Table S21 Energy/electricity burden: % income spend on energy/electricity bill in Arizona and Illinois by different income and ethnic groups (Estimates of increased electricity bill (monthly)). Related to Figure 2.

	Illinois		Arizona							
	Low income	High income	Low income_ Hispanic	Low income_ White	Low income_ Asian	Low income_ African American	High income_ Hispanic	High income_ White	High income_ Asian	High income_ African American
Consumption increase (%)	5.96%	6.33%	9.63%	3.96%	13.00%	6.44%	12.24%	3.12%	8.33%	5.42%
Electricity price $/kWh	0.108	0.108	0.078	0.078	0.078	0.078	0.078	0.078	0.078	0.078
Average hourly electricity usage for low income (kWh)	1.34	1.42	1.15	1.00	0.987208	1.179788	1.43	1.40	1.09	1.32
Daily increase ($)	0.21	0.23	0.21	0.07	0.24	0.14	0.33	0.08	0.17	0.13
Monthly electricity usage increase (kWh)	59.46	66.84	82.61	29.37	95.48	56.53	130.09	32.54	67.86	53.07
**Monthly increase ($)**	**6.41**	**7.21**	**6.43**	**2.29**	**7.43**	**4.40**	**10.13**	**2.53**	**5.28**	**4.13**
Original month bill	107.63	113.91	66.80	57.74	57.19	68.34	82.75	81.20	63.43	76.24
Month income	4666.7	14416.7	3916.67	3916.67	3916.67	3916.67	11833.33	11833.33	11833.33	11833.33
% of Salary	2.31%	0.79%	1.71%	1.47%	1.46%	1.74%	0.70%	0.69%	0.54%	0.64%
**% of Salary (COVID)**	**2.44%**	**0.84%**	**1.87%**	**1.53%**	**1.65%**	**1.86%**	**0.78%**	**0.71%**	**0.58%**	**0.68%**
**% difference**	**0.14%**	**0.05%**	**0.16%**	**0.06%**	**0.19%**	**0.11%**	**0.09%**	**0.02%**	**0.04%**	**0.03%**

Table S22. TOU and non_TOU plan in Arizona (Winter time). Related to STAR Methods.

Plans	Price plan	Observations	Peak hours	On-peak	Off-peak	Unit
TOU	Price plan for residential super peak time-of-use service	18,149,016	3-6pm	$0.1063	$0.0738	$/kWh
	Price plan for residential super peak time-of-use service	758,952	4-7pm	$0.1063	$0.0738	$/kWh
	Experimental plan for residential super peak time-of-use service	931,200	2-5pm	$0.1063	$0.0738	$/kWh
	Standard price plan for residential time-of-use service	18,159,792	5-9am, 5-9pm	$0.0951	$0.0691	$/kWh
Non TOU	Standard price plan for residential service	43,305,504	No	$0.0782	$0.0782	$/kWh

Table S23. Regression Discontinuity Estimates of TOU and Non-TOU Residential Electricity Usage (log) of Covid-19 Policies: Global Results. Related to STAR Methods.

	TOU		Non-TOU	
	School close		School close	
**Covid-19 policy**	0.106	***	0.035	***
	(0.004)		(0.005)	
Weather-related variables	yes		yes	
Month FE	yes		No	
Day-of-week FE	yes		yes	
Holiday FE	yes		No	
Hourly FE	yes		yes	
Account FE	yes		yes	
Observations	9,264,950		10,596,794	
Number of groups	3,246		3,727	
R-square	0.0019		0.0011	

Notes: weather-related control variables includes: temperature (in a restricted cubic spline format), precipitation (linear and quadratic format), air pressure, relative humidity, and wind speed.

Standard errors, clustered by accountid, are in parentheses.
*** Significant at the 1 percent level. ** Significant at the 5 percent level. * Significant at the 10 percent level.

Table S24 Regression Discontinuity Estimates of TOU Residential Electricity Usage (log) of Covid-19 School Closure Mandate: Global Results. Related to STAR Methods.

	white low income		white high income		Hispanic low income		Hispanic high income		other low income		other high income
**Covid-19 policy**	0.072	***	0.102	***	0.117	***	0.130	***	0.107	***	0.122***
	(0.102)		(0.010)		(0.020)		(0.039)		(0.030)		(0.029)
Weather-related variables	yes		yes		yes		yes		yes		yes
Month FE	yes		yes		Yes		yes		Yes		yes
Day-of-week FE	yes		yes		yes		yes		yes		yes
Holiday FE	yes		yes		yes		yes		yes		yes
Hourly FE	yes		yes		yes		yes		yes		yes
Account FE	yes		yes		yes		yes		yes		yes
Observations	782,305		1,376,513		324,391		130,548		175,293		136,413
Number of groups	273		481		113		46		61		49
R-square	0.0013		0.0016		0.0022		0.0022		0.0031		0.0015

Notes: weather-related control variables includes: temperature (in a restricted cubic spline format), precipitation (linear and quadratic format), air pressure, relative humidity, and wind speed.

Standard errors, clustered by accountid, are in parentheses.

*** Significant at the 1 percent level. ** Significant at the 5 percent level. * Significant at the 10 percent level.

Table S25. Energy/electricity burden: % income spend on energy/electricity bill in Arizona and Illinois by different income and ethnic groups for TOU users (Estimates of increased electricity bill (monthly)). Related to STAR Methods.

Winter	**Arizona**							
	Low income_ Hispanic	Low income_ White	Low income_ Asian	Low income_ African American	High income_ Hispanic	High income_ White	High income_ Asian	High income_ African American
On-peak hours (2pm-8pm)								
Consumption increase (%)	18.04%	8.70%	27.92%	16.07%	21.68%	17.90%	14.49%	19.30%
Electricity price $/kWh	0.095	0.095	0.095	0.095	0.095	0.095	0.095	0.095
Average hourly electricity usage for low income (kWh)	1.17	0.97	0.89	1.08	1.48	1.28	0.86	0.69
Daily increase ($)	0.14	0.06	0.17	0.12	0.21	0.15	0.08	0.09
off-peak hours								
Consumption increase (%)	6.42%	4.14%	11.49%	6.41%	7.11%	4.75%	1.61%	10.68%
Electricity price $/kWh	0.069	0.069	0.069	0.069	0.069	0.069	0.069	0.069
Average hourly electricity usage for low income (kWh)	0.98	0.91	0.88	1.01	1.13	1.31	0.81	0.65
Daily increase ($)	0.07	0.04	0.12	0.08	0.09	0.07	0.02	0.08
Total								
Total Daily increase ($)	0.21	0.10	0.28	0.19	0.31	0.23	0.10	0.17
Monthly electricity usage increase (kWh)	6.65	3.11	8.81	5.94	9.55	6.99	3.05	5.28
Orginal month bill	62.32	41.24	73.45	62.77	86.75	76.88	24.29	42.17
Month income	3916.67	3916.67	3916.67	3916.67	11833.33	11833.33	11833.33	11833.33
% of Salary	1.59%	1.05%	1.88%	1.60%	0.73%	0.65%	0.21%	0.36%
**% of Salary (COVID)**	**1.76%**	**1.13%**	**2.10%**	**1.75%**	**0.81%**	**0.71%**	**0.23%**	**0.40%**
**% difference**	0.17%	0.08%	0.22%	0.15%	0.08%	0.06%	0.03%	0.04%

Table S26. Two stage event study estimates of residential electricity of residential electricity Usage (log) of 15 days (Arizona) and 4 days (Illinois). Related to STAR Methods.

	Arizona		Illinois
	School close		School close
**Covid-19 mandates**	0.029	***	0.032
	(0.001)		(0.001)
Weather-related variables	yes		yes
Month FE	yes		No
Day-of-week FE	yes		yes
Holiday FE	yes		No
Hourly FE	yes		yes
Account FE	yes		yes
Observations	5,530,149		8,793,256

Notes: weather-related control variables include temperature (in a restricted cubic spline format), precipitation (linear and quadratic format), air pressure, relative humidity, and wind speed.
Standard errors, clustered by account id, are in parentheses.
*** Significant at the 1 percent level. ** Significant at the 5 percent level. * Significant at the 10 percent level

Table S27. Two stage event study estimates of residential electricity of commercial electricity usage (log) of 15 days (Arizona) and 4 days (Illinois). Related to STAR Methods.

	Arizona		Illinois
	School close		School close
**Covid-19 mandates**	-0.057	***	-0.081
	(0.001)		(0.001)
Weather-related variables	yes		yes
Month FE	yes		No
Day-of-week FE	yes		yes
Holiday FE	yes		No
Hourly FE	yes		yes
Account FE	yes		yes
Observations	15,683,045		8,743,262

Notes: weather-related control variables include temperature (in a restricted cubic spline format), precipitation (linear and quadratic format), air pressure, relative humidity, and wind speed.
Standard errors, clustered by account id, are in parentheses.
*** Significant at the 1 percent level. ** Significant at the 5 percent level. * Significant at the 10 percent level.

Section S1 An analysis of stay-at-home order

In Arizona, starting at 5 pm on March 31st, 2020, the entire state followed the stay-at-home order[73]. Thus, we adopted April 1st (the next day since the stay-at-home order started at 5 pm on March 31st) as the threshold. In Illinois, the entire state entered the stay-at-home order phase with business closed at the same time, which was 5pm on March 21st [64,65]. Thus, the stay-at home thresholds for Illino is March 22nd (the next day since the stay-at-home order started at 5pm on March 21st).

We adopted the same methodology to explore the impact of the electricity consumption from the stay-at home measures. The results are presented below.

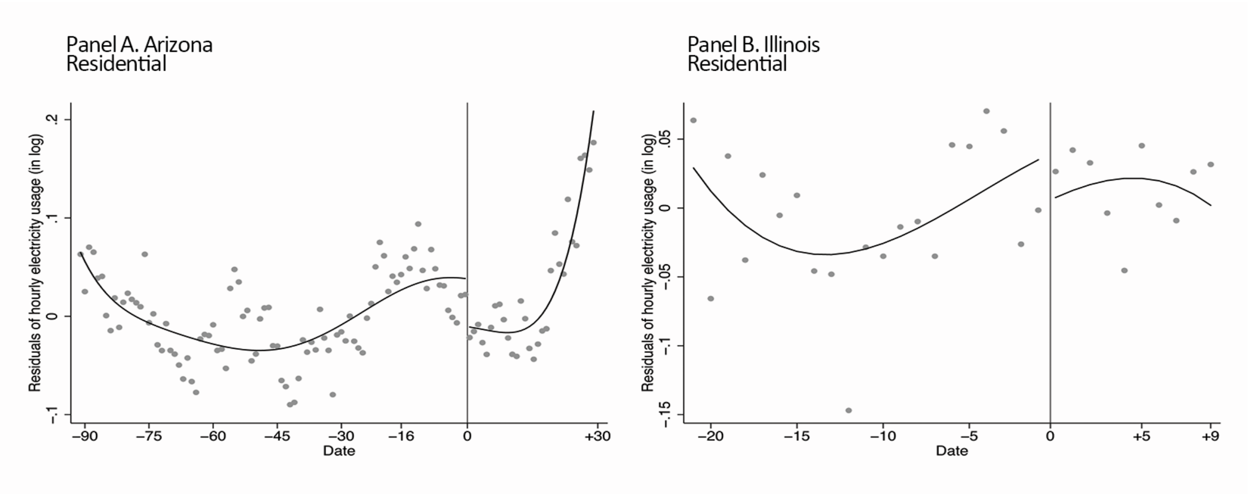

Figure S14. Residential daily averaged hourly electricity consumption percentage change in Arizona and Illinois. Related to STAR Methods. The black circles are daily averaged hourly residuals of log electricity consumption, averaged across all hours of the day (after controlling for covariates, such as weather, the hour of the day, day of the week, the month of the year) for both Arizona and Illinois. The black fitted lines are values obtained from regressing the residuals on the mitigation mandate dummies. Due to the short gaps between the days of these two mandates, we adopt a sixth-order polynomial on the date for the stay-at-home mandate in Arizona, and a third-order polynomial on the date for the stay-at-home mandate in Illinois. In both states, the stay-at-home orders were enforced at 5pm so we use the next day as the threshold, while school-close orders were enforced in the morning so we use the same day as the threshold. We conducted a robustness check where we set the threshold of stay-at-home order in AZ as the same day when stay-at-home order was enforced and another robustness check dropping that day.The polynomial orders are chosen by the Bayesian Information Criterion (BIC), and we also reported results for other polynomial orders in Supplemental Table 26.

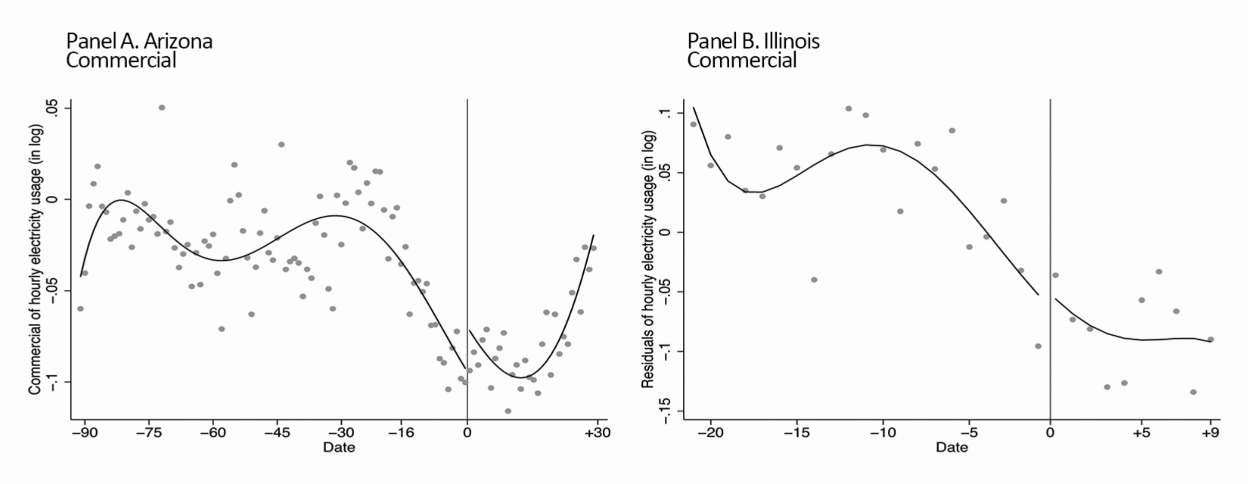

Figure S15. Commercial daily averaged hourly electricity consumption percentage change in Arizona and Illinois. Related to STAR Methods. Notes: The black circles are daily average hourly residuals of log electricity consumption (after controlling for covariates, such as weather, hour of the day, day of the week, and month of the year) for both Arizona and Illinois, averaged across all hours of a day. The black lines are the fitted polynomial to model the nature of the treatment through the strategy of regressing the residuals on these mitigation measure dummies. Due to the different durations of the time window of the two states, to avoid overfitting, we adopt a sixth-order polynomial on the date for stay-at-home mandate in Arizona, and we adopt a fifth-order polynomial on the date for stay-at-home mandate in Illinois. The polynomial orders are chosen by the Bayesian Information Criterion (BIC).

Table S28. Regression Discontinuity Estimates for the Stay-at-home Orders: Global Polynomial Results (Residential and Commercial Sector). Related to STAR Methods.

Polynomial order	3	4	5	6	7	8	BIC
**AZ_residential**	-0.124***	-0.098***	-0.057***	-0.048***	-0.038***	0.002***	-0.057***
	(0.003)	(0.003)	(0.003)	(0.003)	(0.003)	(0.003)	(0.003)
Observations	19,998,526	19,998,526	19,998,526	19,998,526	19,998,526	19,998,526	19,998,526
Number of groups	7,004	7,004	7,004	7,004	7,004	7,004	7,004
R-square	0.00111	0.0021	0.0026	0.0027	0.0028	0.003	0.0021
**IL_residential**	-0.034***	-0.001***	0.018***	0.034***	0.084***	0.086***	-0.034***
	(0.002)	(0.002)	(0.002)	(0.002)	(0.002)	(0.002)	(0.002)
Observations	30,283,001	30,283,001	30,283,001	30,283,001	30,283,001	30,283,001	30,283,001
Number of groups	40,771	40,771	40,771	40,771	40,771	40,771	40,771
R-square	0.001	0.001	0.001	0.002	0.002	0.002	0.001
**AZ_commercial**	-0.038***	-0.009***	0.033***	0.026***	0.035***	0.043***	-0.009***
	(0.002)	(0.002)	(0.002)	(0.002)	(0.002)	(0.002)	(0.001)
Observations	37,564,057	37,564,057	37,564,057	37,564,057	37,564,057	37,564,057	37,564,057
Number of groups	14,271	14,271	14,271	14,271	14,271	14,271	14,271
R-square	0.001	0.001	0.002	0.002	0.002	0.002	0.002
**IL_commercial**	-0.028***	0.030***	0.011***	0.040***	0.048***	0.048***	0.011***
	(0.002)	(0.002)	(0.002)	(0.002)	(0.002)	(0.002)	(0.002)
Observations	30,114,636	30,114,636	30,114,636	30,114,636	30,114,636	30,114,636	30,114,636
Number of groups	40.757	40,757	40,757	40,757	40,757	40,757	40,757
R-square	0.001	0.001	0.001	0.001	0.001	0.001	0.001
